# Progressive applications of synthesized isatin-based reactive dye: fabrics dyeing, corrosion inhibition, and antimicrobial activity

**DOI:** 10.1038/s41598-025-30978-3

**Published:** 2025-12-23

**Authors:** Mervat El-Sedik, Saadia Abd Elmegied, Tarek Aysha, Amal M. Abdel-karim, Bassant Gaber, Safia A. Mahmoud

**Affiliations:** 1 Dyeing, Printing and Textile Auxiliaries Department, Textile Research and Technology Institute, National Research Centre, 33 EL Buhouth St., Dokki, Giza, 12622 Egypt; 2 Physical Chemistry Department, National Research Centre, 33 El Bohouth St, Dokki, Giza, Egypt; 3 Faculty of Biotechnology, October University of Modern Sciences and Arts (MSA), 6th of October City, Egypt

**Keywords:** Isatin, Reactive dye, Dyeing, Corrosion inhibitor, Antimicrobial, Molecular docking, Chemistry, Materials science

## Abstract

A new heterobis-reactive dye, monochlorotriazine/sulphatoethylsulphone bearing an isatin moiety (MCT-SES dye), was synthesized and identified using NMR, mass spectra, and elemental analysis. The prepared reactive dye showed an absorption band at 543 nm. The dyeing properties on cotton and wool fabrics were investigated at different concentrations and pH levels, showing excellent dye affinity toward both fabrics. The dyed fabrics exhibited excellent wash fastness, rubbing fastness, perspiration fastness, and good light stability. In 1.0 M hydrochloric acid solution, the isatin-based MCT-SES dye exhibited unique corrosion inhibition performance for carbon steel (CS). Energy dispersive X-ray spectroscopy (EDX), scanning electron microscopy (SEM), electrochemical impedance spectroscopy (EIS), and potentiodynamic polarization (PDP) were the methods used to assess the inhibitory efficacy and development of a protective film on the steel surface. With increasing dye concentration, the corrosion inhibition efficiency increased, reaching a maximum of 98.64% at 500 ppm. The excellent performance was attributed to strong adsorption and the formation of a dense, protective barrier film on the steel surface. The MCT-SES dye demonstrated strong antibacterial action against *P. aeruginosa* (23 ± 0.23 mm) and *E. coli* (20 ± 0.34 mm). The inhibition of the bacteria growing on the surface of the dyed fabrics was investigated by scanning electron microscope (SEM), which proves the high affinity of the dyed fabric against bacteria growth. Molecular docking simulations and the binding interaction of the dye structure with the bacteria protein were investigated.

## Introduction

 Reactive dyes are the second most widely used class of synthetic dyes; they are used to color 50% of the textiles and fabrics used worldwide. Cellulosic fibers and some silk and wool fibers are the primary materials dyed using reactive dyes^1–6^. Reactive dyes are water-soluble and contain reactive groups that bond covalently with fiber molecules. Reactive dye has the benefits of strong application, excellent color fastness, and vibrant color^7,8^. Bifunctional reactive dyes are increasingly popular due to their higher fixation efficiency compared to dyes containing a single reactive group. They utilize sulphato-ethylsulphone (SES) and monochlorotriazine (MCT) reactive groups, which enhance their physicochemical and dying properties^8–10^. Monochlorotriazine is a commonly used reactive dye that contains a distinctive halogenated heterocyclic reactive group connected to the chromogenic system. This dye forms covalent bonds with fibers in alkaline conditions. Triazine reactive dyes can react with both the fiber and water, creating a substance that can affect color fastness on fabric. The three main variables that affect reactive dye affinity and fixation are temperature, salt and alkali concentrations, and liquid ratio in the dyebath^1,3,11^. The sulphato- ethylsulphone group is a highly reactive group that is frequently used in the dyestuff industry. Its high reactivity makes it effective for dyeing cotton fabric with low liquor ratio and cold pad-batch dyeing methods at lower temperatures. In an alkali conditions, the sulphato- ethylsulphone group is convert to the hydrophobic vinyl sulfonyl group, which may reduce the dye’s solubility in water^12^.

Isatin, also known as 1 H-indole-2, 3-dione, is a compound with significant biological and pharmacological properties. It is commonly used as a precursor in drug synthesis and in the creation of various heterocyclic compounds like quinolones and indoles^11,13^. Isatin is a versatile heterocyclic compound composed of two rings, one six-membered and the other five- membered. It exhibits a diverse array of biological effects, including antioxidant, anti-inflammatory, antimicrobial (against bacteria, viruses, and fungi), anti-tuberculosis, anticancer, anticonvulsant, anti-HIV, and more. In addition to its biological properties, isatin finds applications in various industrial fields, such as dyes manufacturing and corrosion inhibition, owing to its adaptable reactivity^14–17^.

Metal corrosion poses a significant industrial and economic challenge, leading to metal deterioration and increase maintenance costs. The use of organic inhibitors containing nitrogen and oxygen donor atoms is one of the most efficient and cost-effective compound due to their excellent inhibition performance.

However Isatin (1 H-indole-2, 3- dione) and its derivatives have shown promising corrosion inhibition performance, particularly for iron in acidic media. Their conjugated structure and reactive carbonyl groups facilitate strong adsorption on metal surfaces, leading to the formation of a protective layer that minimizes dissolution. Structural modifications, particularly heterocyclic derivatives, can enhance their inhibition efficiency. Isatin also contains benzoyl rings and amino functional groups, which play a key role in the inhibition and adsorption processes^18^. Various heterocyclic inhibitors, including salycilidene isatin hydrazine sodium sulfonate^19^, triazine ring- containing isatin compounds^20^, Carbamothiol oxalamide derivatives^21^ and Quinoxalinosulfonamide hybrid-bearing theophylline^22^. Hydrazones are a diverse group of chemical compounds with the structure R₁R₂C = NNR₃R₄, formed by condensation of isatin with hydrazine. Medicinal chemists are interested in these compounds because they contain an azomethine group (-NH-N = CH-) linked to a carbonyl group. Hydrazones have strong biological activities and have been explored for therapeutic use, showing effects such as anti-inflammatory, anticonvulsant, antifungal, cytostatic, and cytotoxic properties against bacteria, microorganisms, and tumors^11^. Cotton textiles are prone to microbial attack due to their large surface area and moisture-absorbing properties, creating ideal conditions for microbial growth. This can lead to unpleasant odors, product damage, skin contamination, allergic reactions, and potential health risks from the spread of organisms^23^. Antimicrobial dyes applied to textiles have been proven to prevent bacterial growth, thereby preventing a decline in fabric strength and quality, as well as reducing the occurrence of stains, odors, and health problems caused by microbes.

The medical field and various aspects of everyday life can both reap the advantages of antibacterial textiles^24^. Protein-ligand docking is a commonly employed method to study the interaction between dyes and bacterial enzymes and assess their antimicrobial properties. Molecular docking helps in optimizing and understanding the significance of ligand-protein interactions, allowing for the prediction of specific amino acid residues in bacterial enzymes that interact with dye molecules^25,26^. This study aims to develop a new reactive dye for cotton and wool fabrics. Additionally, the study aims to investigate the dye as an eco-friendly corrosion inhibitor for carbon steel in a 1 M hydrochloric acid solution by evaluating its inhibitory efficiency. Potentiodynamic polarization (PDP), electrochemical impedance spectroscopy (EIS), and scanning electron microscopy (SEM & EDX) were used to assess the corrosion protection of carbon steel in a 1 M HCl solution. Furthermore, the study will also explore the antibacterial effects of the dye and conduct molecular docking investigations.

## Materials and instruments

### Materials

H-acid (8-amino-1-naphthol-3,6-disulfonic acid), cyanuric chloride (2,4,6-Trichloro-1,3,5-triazine), isatin (99.0%), 1-aminobenzene-4-β-sulphatoethylsulphone, and hydrazine hydrate were purchased from Sigma-Aldrich(Germany). The synthesis was carried out using highly pure solvents. Analytical-grade hydrochloric acid (37%) from Merck was used without additional purification. El-Mahalla El-Kobra Company, Egypt provided (160 g/m^2^, Ne 120/2, 69 End/cm, 22 Picks/cm) bleached cotton fibers as well as bleached wool fabrics. Fabrics were further cleaned to remove any contamination by agitating them for 30 min at 80 °C in a water bath with 2 g/L of nonionic detergent. Thin-layer chromatography (TLC) was performed using a Kieselgel 60 F254 device from Merck, Germany.

### Instruments

Stuart melting point apparatus, UK was used to determine the compounds’ melting points. An EA 1108 apparatus from Fisons Instruments, USA was used for elemental analysis. A Bruker DMX-400 spectrometer, Germany operating at 400 and 101 MHz was used to record the^1^H and^13^C NMR spectra. Mass spectra were obtained using a Varian MAT CH-5 spectrometer (70 eV), Germany. UV/visible spectra were measured using a Shimadzu UV-2401 PC UV/Vis spectrophotometer, Japan. Absorption spectra in the ultraviolet-visible range of 200–700 nm in dimethylsulfoxide (DMSO) as a solvent were measured with an Ultra Scan PRO spectrophotometer (Hunter Lab) Germany, equipped with a D65 illuminant and a 10° standard observer. The visible color strength (K/S) of the dyed fabric was measured using Hunter Lab UltraScan PRO spectrophotometer (USA) equipped with an illuminant D65 and a 10-degree standard observer was used to take the color measurements.

### Synthesis of dye

**Scheme 1:**
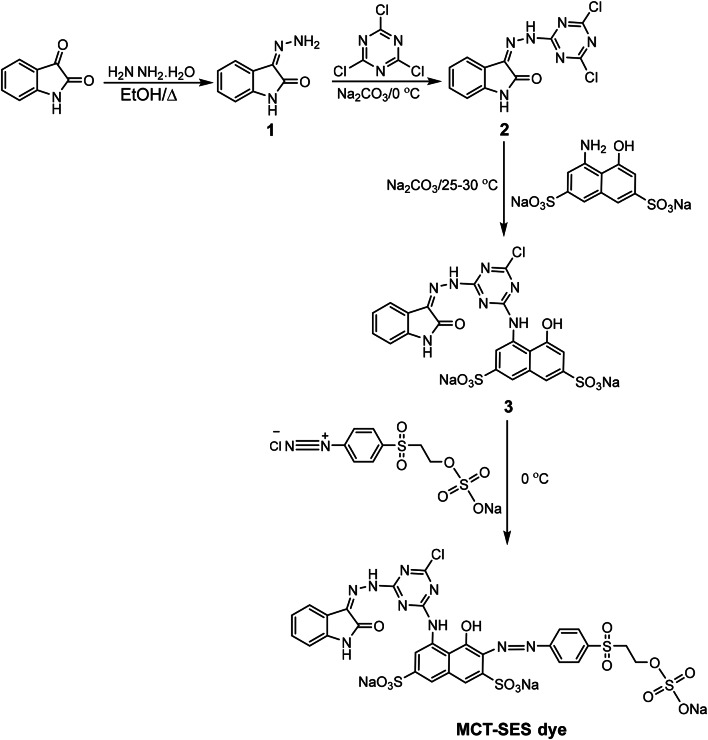
Synthetic pathway for MCT-SES dye.

The MCT-SES dye was prepared in four steps, following the process outlined in Scheme 1. Intermediates 1 (isatin hydrazone), 2 (dichloro triazinyle derivative), and 3 (monochloro triazinyle derivatives) are essential intermediates in the synthesis of the MCT-SES dye and were synthesized using a method described in previous studies^6,27–30^.

### Synthesis of reactive dye (MCT/SES)

#### Synthesis of isatin hydrazone (1)

1.47 g (0.01 mol) of isatin (indole-2, 3-dione) was dissolved in 20 mL of ethanol, and then 0.015 mol of hydrazine hydrate (99%) was added. The mixture was stirred, heated on a water bath for 10 min, and subsequently cooled in the fridge for 3 h. The yellow crystalline solid that formed was filtered, washed with cold water and ethanol, and dried. The product was purified by recrystallization from chloroform, resulting in an 80% yield of the product with a melting point of 220 °C ^27^.

#### Synthesis of (*Z*)−3−2-(4,6-dichloro-1,3,5,triazin-2-yl)hydrazono)indolin-2-one(2)

To a mixture of cyanuric chloride (2, 4, 6-Trichloro-1, 3, 5-triazine) (0.05 mol), Na_2_CO_3_ (0.1 mol), and 100 mL anhydrous THF, isatin hydrazone (1) (0.05 mol) that had been dissolved in 50 mL of tetrahydrofuran (THF) was added dropwise. Then the mixture had been stirred for about five hours at 0 °C till the starting material disappeared monitoring with Thin layer chromatography (TLC) using an eluent system of n-Hexane: Ethyl acetate (3:1, v/v). After filtering out the inorganic residue, compound 2 (yield: 75%) was isolated from the filtrate by precipitated using 50 mL cold water^30^.

#### Synthesis of sodium (*Z*)−4-((4-chloro-6-(2-(2-oxoindolin-3-ylidene) hydrazinyl)−1, 3, 5-triazin-2-yl) amino)−5-hydroxynaphthalene-2, 7-disulfonate (3)

In a 200 mL round flask, 4.0 g (0.01 mol) of H-acid was added to 3.8 g (0.01 mol) of compound 2 in 50 mL of THF at a pH range of 5–5.5.5 using a 10% aqueous solution of Na_2_CO_3_, at a temperature range of 25–30 °C. Compound 3 was formed in the solution after the reaction mixture was stirred for approximately four hours. The progress of the reaction was monitored using TLC with an eluent system consisting of n-PrOH: i-BuOH: EtOAc: H2O (2:4:1:3, v/v) ^6^.

#### Synthesis of sodium 2-((4-((E)-(8((4-chloro-6-(2-((*Z*)−2-oxoindolin-3-ylidene) hydrazinyl)−1,3,5-triazin-2-yl)amino)−1-hydroxy-3,6-disulfonatonaphthalen-2yl)diazenyl) phenyl)sulfonyl)ethyl sulfate (MCT-SES dye)

To perform the diazotisation process of 1-Aminobenzene-4-*β*-sulphatoethylsulphone, 2.8 g (0.01 mol) was dissolved in 2.5 mL (0.015 mol) of 37% hydrochloric acid. After cooling the solution in an ice water bath, 1 g (0.014 mol) of NaNO_2_ was gradually added over the course of 20 min in 25 mL of distilled water. For half an hour, the reaction mixture was stirred. The diazonium salt solution was created and the excess of nitrous acid was removed using a very small traces of sulfamic acid which monitored using iodide-starch paper. As the coupling agent, the resultant diazonium salt was then progressively added to a cooled solution of compound 3. After stirring the mixture for seven more hours at 0 °C, it was left to agitate overnight at ambient temperature. After filtering, washing with acetone, and drying at 40 °C, 2.8 g of MCT-SES dye (78%) with a melting point higher than 300 °C was obtained.

^1^H-NMR: (DMSO-d^6^, 300 MHz): δ = 4.9 (t, 2 H, CH_2_), 5.08(t, 2 H, CH_2_), 6.8–8.9(m, 11 H, aromatic), 9.5(s, 1H, OH), 10.03 (s, 1H, NH), 10.26 (s, 1H, NH), 10.86 (s, 1H, NH).

^13^C-NMR: (DMSO-d^6^, 125 MHz): 58.3, 61.2, 108.1, 115.9, 117.7, 119.5, 120.2, 120.9, 123.9, 124.5, 125.3, 127.1, 128.6, 129.4, 129.8, 130.4, 131.2, 133.1, 135.7, 137.6, 138.7, 141.1, 143.2, 144.8, 157.7, 168.6, 169.8, 170.5, 180.2.

Mass spectra: m/z (%): 951.21 ([M + H]^+^, 100), (MW 950.19 g mol^- 1^). Anal. calcd. For C_29_H_19_ClN_9_Na_3_ O_14_S_4_: C (36.66%) H (2.02%) N (13.27%) S (13.50%); found: C (37.04%) H (2.18%) N (13.19%) S (13.20%).

### Dyeing procedure

#### Dyeing of cotton fabrics

**Fig. 1:**
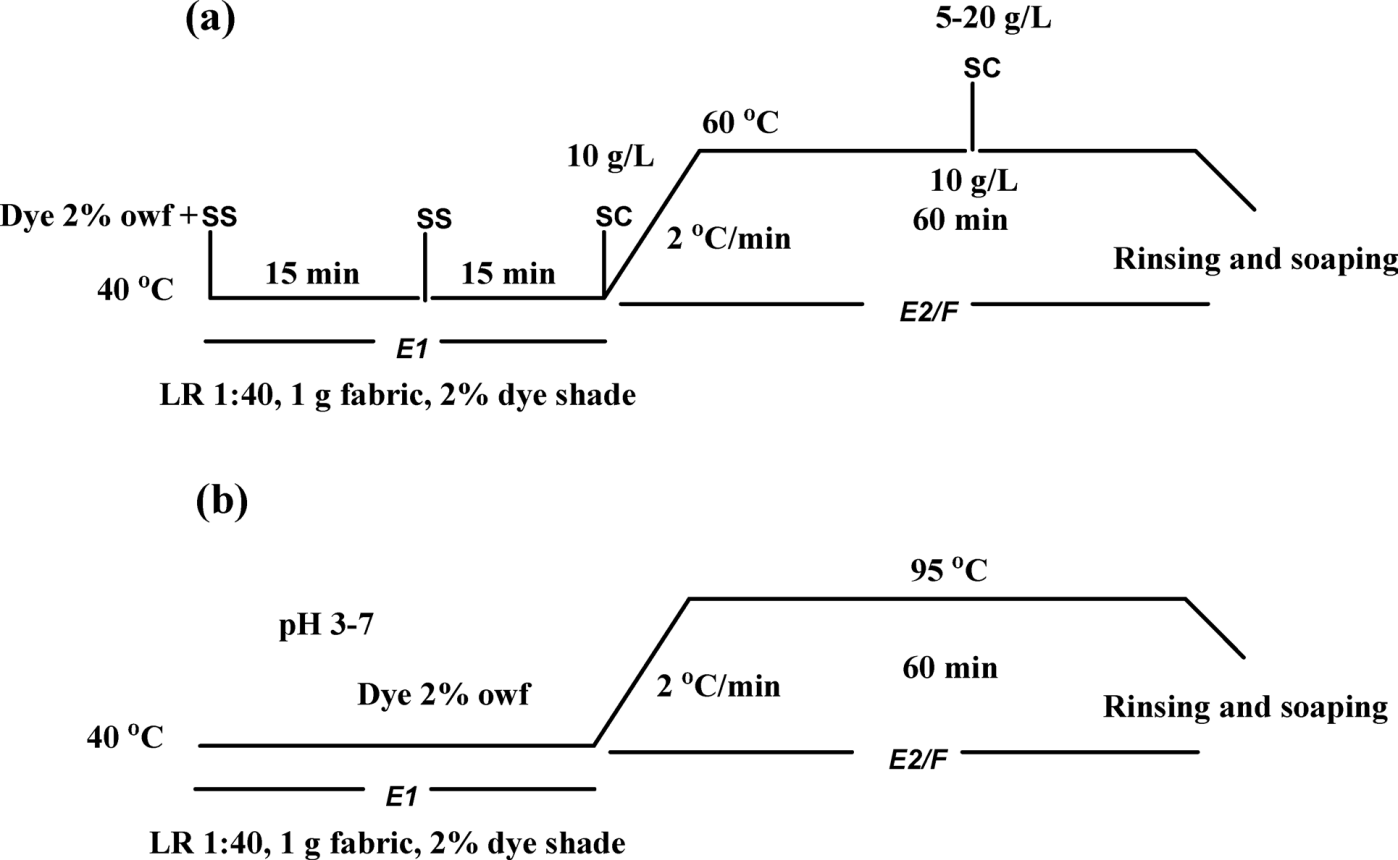
Dyeing diagram illustrating the process of dyeing cotton fabrics (**a**) and dyeing of wool (**b**).

The MCT-SES dye was used in a dyeing application with a liquor ratio of 1:40 (w/v). Sodium sulphate (SS) was added at concentrations of 20–40−60 g/l, followed by varying amounts of sodium carbonate (SC) (5–20 g/L) to adjust the dye bath alkalinity. Different dye concentrations (1–5%) were also tested to observe their impact on dye exhaustion and fixation on cotton fabric. The dyeing process involved primary exhaustion (%*E1*) at 40 °C for 30 min, followed by fixation in sodium carbonate at 60 °C for 60 min for recoding the secondary exhaustion (%*E2*) as shown in Fig. 1(a). After rinsing, the dyed samples were extracted with 50% aqueous DMF at boiling for 15 min to determine the fixation (%*F*) and total fixation (%*T*) of the dye on the cotton fabric through covalent bonding^1,31^.

#### Dyeing of wool fabrics

The fabric wool was dyed using the synthesized reactive dye (MCT-SES dye) at a liquor ratio of 1:50 with a dye concentration of 2% owf. The dyeing process was conducted at pH levels ranging from 3 to 7 to investigate the effect of dye concentration varying from 1% to 5%. The dyeing temperature started at 40 °C and gradually increased to a boil over 30 min, followed by dyeing at a boil for an additional 60 min as shown in Fig. 1 (b). After dyeing, the samples were rinsed thoroughly with a nonionic detergent solution (2 g/L), and then rinsed with cold water and air-dried^32^.

#### Dyeing measurements

A Shimadzu UV-2401PC UV-visible spectrophotometer was used to measure and test the dye exhaustion and fixation on cotton and wool fabric using a spectrophotometric method at the λmax value. Equation 1 was utilized to determine the percentage of dye bath exhaustion (*%E*) by creating a calibration curve with known dye concentrations (g/L):

1 $$\rm \%E=[1-C_2/C_1] \times 100$$

The concentrations of dye before and after dyeing are represented as C1 and C2, respectively. Using the light reflectance approach and the Kubelka- Munk Eq. 2, the K/S value of the dyed materials was determined^33^. The following formula was used to calculate the colored fabrics’ reflectance (R):

2 $$\rm K/S=(1-R)2/2R$$

where K is the absorption coefficient, S is the scattering coefficient, and R is the decimal percent of the dyed fabric’s reflection.

The dye fixation was recorded using to spectrophotometric method by boiling the dyed fabric in DMF/water 1:1 (v/v) using liquor ratio 1:40 for 15 min using Eq. 3.

3 $$\rm \%F=\frac{C_1-C_2-C_3} {C_1-C_2} \times 100$$

where, C_1_ and C_2_ are the dye concentration before and after dyeing and C_3_ the residual of dye concentration after boiling in DMF/water 1:1 (v/v).

### Fastness testing

After dyeing with a 2% shade (owf), cotton and wool samples washed with 2 g/l nonionic detergents at 80 °C for 15 min. The dyed samples were evaluated following standard ISO testing procedures (Methods of Tests for Color Fastness of Textiles and Leather, 1990). Wash fastness was assessed using the visual ISO grey scale for color change [ISO 105-C02 (1989)], and fastness to sweat was evaluated using ISO 105-E04 (1989). Xenon arc light fastness was tested according to ISO 105-B02.

### Corrosion inhibition

#### Electrochemical measurements

Carbon steel (CS) samples with 2.68% C, 0.36% Si, 0.24% Cr, and 96.72% Fe were used in electrochemical testing as the working electrode. The specimens, measuring 1 × 2 × 0.5 cm were polished with various emery paper grades, cleaned with acetone, rinsed with double-distilled water, and then dried. After that, the electrodes were immersed in 1 M HCl test solutions without and with different concentrations of MCT-SES dye (100, 200, 300, 400, and 500 ppm). An Autolab Potentiostat/Galvanostat PGSTAT 302 N was used in a three-electrode Pyrex glass cell arrangement for the studies. The working electrode had an exposed surface area of 1 cm^2^, the reference electrode was silver/silver chloride (Ag/AgCl), and the counter electrode was platinum.

#### Potentiodynamic polarization (PDP)

The inhibition efficiency of MCT-SES dye at different concentrations was evaluated by potentiodynamic polarization after the system reached a steady open-circuit potential (OCP). Measurements were taken within a specific potential range at a scan rate of 1 mVs^− 1^. The Nova 1.11software’s automated fitting tools were utilized to identify the linear regions of the cathodic and anodic polarization curves, showing a clear logarithmic relationship between potential and current density. This allowed for the estimation of Tafel parameters. The Tafel extrapolation method was employed to calculate the inhibition efficiency (IE%) using Eq. 4.

4 $$\rm \:IE{\%}=\frac{{i}_{o}-{i}_{i}}{{i}_{0}}\times 100$$

where, i_o_, i_i_ are the current densities without and with MCT-SES dye inhibitor, respectively.

#### Electrochemical impedance spectroscopy (EIS)

A 10.0 mV peak-to-peak sinusoidal signal was used to measure AC impedance responses at open circuit potential (OCP) in the frequency range of 0.01 Hz to 100 kHz. The measurements included phase shift (θ) and impedance (Z). Each test was repeated three times at room temperature to ensure accuracy.

### Surface morphology

Surface morphology of carbon steel samples in 1 M HCl solution, without and with 500 ppm of MCT-SES dye, was analyzed using Scanning electron microscopy (SEM) JSM-6510 microscope equipped with an energy dispersive X-ray spectrometer (EDX) (Quantax75).

### Antimicrobial evaluation

#### Microorganism’s preparations and conditions

Five microbial pathogens of public health significance were selected to evaluate the antimicrobial potential of synthesized organic materials, representing a diverse range of microorganisms. These included two Gram-negative bacterial species, *Escherichia coli* and *Pseudomonas aeruginosa*, three Gram-positive bacterial species, *Staphylococcus aureus*, *Enterococcus faecalis*, and the fungal species *Candida albicans*. These organisms were chosen due to their clinical and environmental relevance. For bacterial species, fresh cultures were grown in Trypticase Soy Broth (TSB) at 37 °C overnight and harvested during the mid-logarithmic phase. The fungal species were cultivated in Sabouraud Dextrose Broth (SDB) under the same incubation conditions. After incubation, all microbial cultures were centrifuged at 6000 rpm for 15 min to separate the cells. The resulting pellets were washed twice with phosphate-buffered saline (PBS) to eliminate residual macromolecules and media components. The prepared microbial suspensions were standardized to 5.0 × 10^7^ CFU/mL for subsequent experiments, ensuring uniformity across all assays^34,35^.

#### Inhibition zone assay

The antimicrobial activity of the blank and dyed textile samples (cotton and wool) and the MCT-SES dye was evaluated using the agar well diffusion method. Briefly, freshly cultured pathogenic microorganisms, *E. coli*, *P. aeruginosa*, *S. aureus*, *E. faecalis*, and *C. albicans*, were uniformly spread on Mueller-Hinton agar plates (for bacteria) and Sabouraud dextrose agar plates (for *C. albicans*), using sterile cotton swabs to achieve a lawn of growth. The specific pieces, 2.5 × 2.5 cm of blank cotton and wool, and dyed cotton and wool, were applied onto the agar. Wells of uniform diameter 6 mm were aseptically punched into the agar surface, and each well was loaded with either 100 µL of pure MCT-SES dye solution or blank textile extract as a control. The plates were incubated under appropriate conditions: at 37 °C for bacterial species and 28 °C for fungal species (*C. albicans*). The diameters of the inhibition zones (DIZ) around each disc or well were measured in millimeters (mm) using a calibrated digital caliper, providing a quantitative measure of antimicrobial efficacy^36^.

#### Estimation of cell viability

The antibacterial performance of dyed wool and cotton textiles was assessed quantitatively using a broth dilution viability assay. The bacterial suspension was prepared in sterile distilled water, yielding an initial concentration of 6.4 × 10⁶ CFU/mL. Aliquots (0.1 mL) of suitable dilutions of each bacterial solution were inoculated onto nutrient agar plates. Subsequent serial tenfold dilutions of the bacterial cultures were executed. The suspensions were then prepared in repeated tenfold dilutions.For the cell viability experiment, fabric samples of 2.5 × 2.5 cm were submerged in the bacterial solution and incubated under identical conditions. At specified time intervals (5, 15, 30, 45, 60, and 90 min), aliquots were serially diluted and plated to ascertain viable cell counts^37^.

#### Visualization of bacterial damaging using scanning electron microscope (SEM)

SEM analysis was conducted to visualize the morphological interaction between the tested bacterial strains and the textile substrates (cotton and wool) before and after the antimicrobial MCT-SES dye. For this, fabric samples 2.5 × 2.5 cm were incubated with *P. aeruginosa* and *E. faecalis* bacterial suspensions at approximately 10⁶ CFU/mL concentration in sterile nutrient broth. The incubation was carried out at 37 °C for 24 h under static conditions to allow bacterial adhesion and colonization. Following incubation, the samples were gently rinsed with sterile phosphate-buffered saline (PBS) to remove non-adherent bacteria. The fabrics were then fixed using 2.5% glutaraldehyde in PBS for 2 h at 4 °C. After fixation, samples were dehydrated through a graded ethanol series (30%, 50%, 70%, 90%, and 100%), each for 10 min. The dehydrated specimens were air-dried, mounted on aluminum stubs using carbon tape, and sputter-coated with a thin layer of gold to enhance conductivity. The samples were examined using a scanning electron microscope TSCAN VEGA 3, Czech Republic at an accelerating voltage of 20 kV. Images were captured at various magnifications to assess the degree of bacterial adhesion and morphological alterations. Comparisons were made between the untreated (blank) and MCT-SES-dyed textile samples to evaluate the antimicrobial surface effects.

### Molecular docking evaluation

#### Compound preparation

The prepared dye was optimized using Avogadro 1.2.0 molecular modeling software with the MMFF94 force field due to their organic nature^38^. The active sites of the proteins were identified using CB-Dock 2. Molecular docking studies were then performed using AutoDock Vina to determine how the compounds bind to the protein targets and their affinities^39^. The docked poses were visualized and analyzed using Biovia Discovery Studio (Dassault Systèmes BIOVIA).

#### Protein preparation

The crystal structures of the following proteins were retrieved from uniport and prepared using AutoDock Tools 1.5.7^39^. A. baumannii-PBPA-P02918, P. aeruginosa-mrcA-Q07806, P. aeruginosa OPR-Q51487, and S. aureus-gyrB-Q6GKU0, S. aureus-pbp-P07944, and Str.mutans-gyrB-Q8CQK4. All of them were selected due to their direct relation with bacterial inhibition mechanism, hydropathy added, Kollman charges assigned, and pdbqt output saved to further processing. The active sites of the prepared proteins were detected using CB-Dock 2^39^.

## Results and discussions

### Synthesis of reactive dye (MCT/SES)

The MCT-SES dye was synthesized according to Scheme 1. Intermediate 1 was prepared by condensing isatin with hydrazine hydrate in ethanol, resulting in yellow crystals with a good yield^6,27^. The reaction between intermediate 1 and cyanuric chloride in dry THF under controlled pH and temperature conditions resulted in the formation of dichloro triazinyle derivative 2, which was then neutralized with sodium carbonate^29^. In the presence of sodium carbonate, at a temperature range of 25–30 °C and a pH of 5–5.5.5, the second chlorine atom in compound 2 was substituted with an H-acid through electrophilic substitution to produce monochloro triazinyl derivative 3. Compound 3 was subsequently combined with the diazonium salt of 1-Aminobenzene-4-*β*-sulphatoethylsulphone to form the MCT-SES dye. The structure of the synthesized dye was confirmed using NMR. The ^1^HNMR spectra showing two triplet beaks at 4.50 and 5.08 δ corresponding to the aliphatic methylene groups and aromatic protons recorded in the range 6.80–8.90 δ, a three singlet beaks at 10.03, 10.26 and 10.86 δ which are corresponding to the NH proton, the NH located in the H-acid appears at 10.03 while the hydrazide NH appears at longer chemical shift at 10.86 due to the deshielding effect of triazine ring while the isatin NH recorded at 10.26 δ. The OH proton appears at 9.5 δ. The molecular weight of the prepared dye was confirmed by mass spectra.

### Absorption spectra

**Fig. 2:**
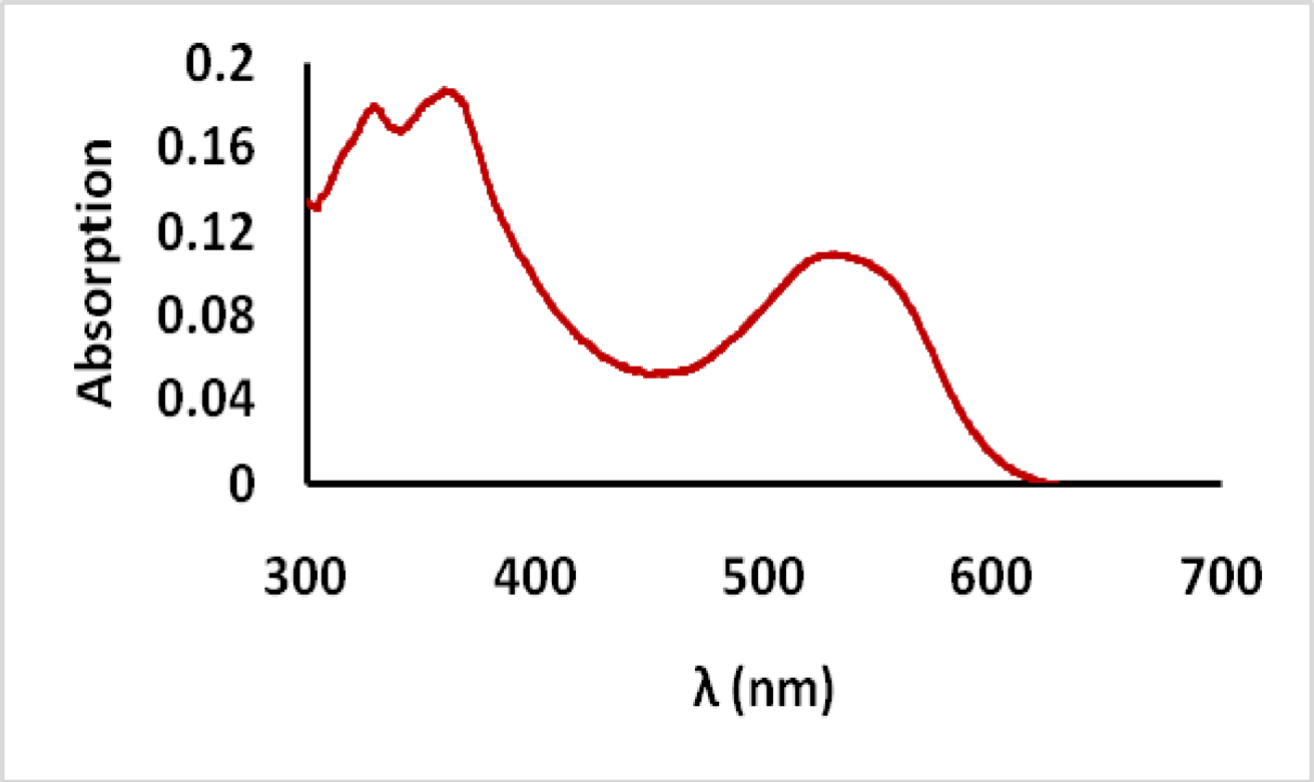
Absorption spectra of the synthesized dye (MCT-SES dye) in DMSO, 20 µMol.

The absorption spectrum of the prepared dye (MCT-SES dye) was recorded using spectrophotometer as shown in Fig. 2 which disolved in DMSO, 20 *µ*Mol concentration. The spectra showing a significant two vibrionic absorption bands at 325, 355 nm which corresponding to the isatin moiety, while a clear absorption band appear at 543 nm corresponding to the H acid bearing the azo dye chromophoric moitey. The molar absorbativity coeffient was recorded (9500 L. M^− 1^. Cm^− 1^).

### Dyeing application

**Scheme 2:**
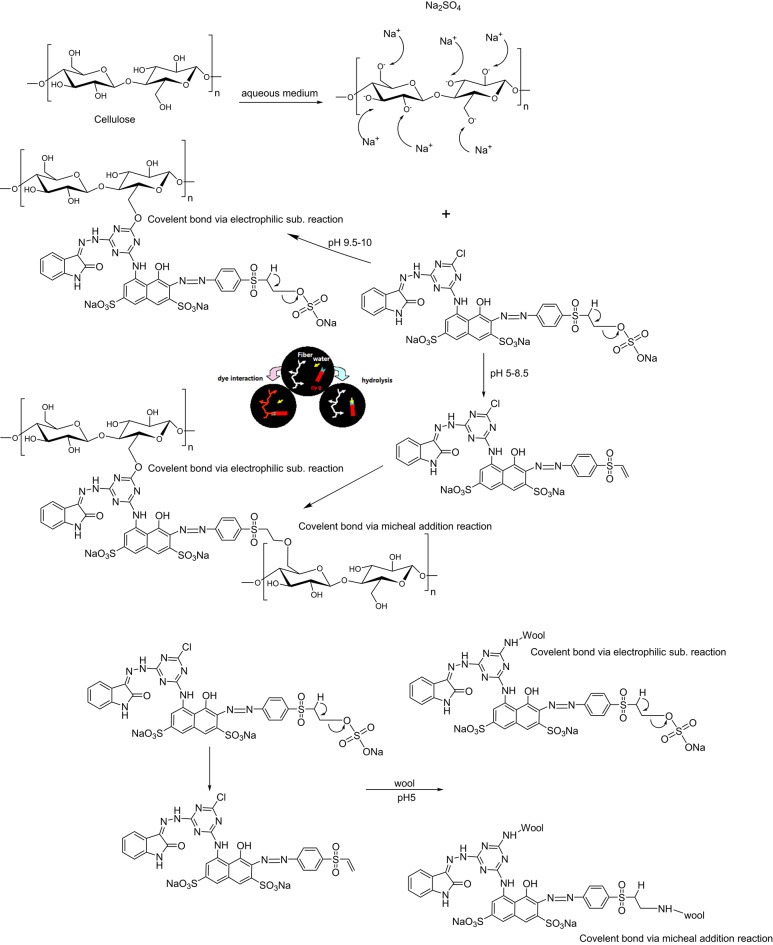
The dyeing mechanism of the MCT/SES reactive dye with cotton and wool fabrics.

 The dyeing application mechanism is presented in Scheme 2. It is known that cellulosic materials acquire negative surface charges upon dipping in water^40^. Thus, it is conventional to add inorganic salt (sodium sulphate) to cover located negative charges formed in the surface of cellulosic fibers due to the ionization of the hydroxyl groups in the glucose units to avoid the mutual electrostatic repulsions between the surface of the fabrics and the dye molecules. Therefore, as shown in Scheme 2, the first phase of dyeing reveals the formation of an electrical double layer between the surfaces of the fabric and the sodium cations, thus suppressing the repulsion to allow dye exhaustion. In the second phase, and while the sodium salt to active sites of the fabrics, proton abstraction takes place by virtue of the hydroxyl anions of the fabrics in case of cotton and with amino group in the case of wool allowing dye fixation via nucleophilic addition (VS-type reactive dyes) which formed vi *β* elimination reaction of sulphatoethylsulphone forming the vinyl form (VS-type) or nucleophilic substitution (MCT-type reactive dyes) onto cotton fabrics and by both mechanism for both fabrics, we cannot neglect the possibility of the hydrolysis process due to the interaction of the reactive site on the dye structure with water.

### Dyeing of cotton fabrics

#### Effect of salt concentration

**Fig. 3:**
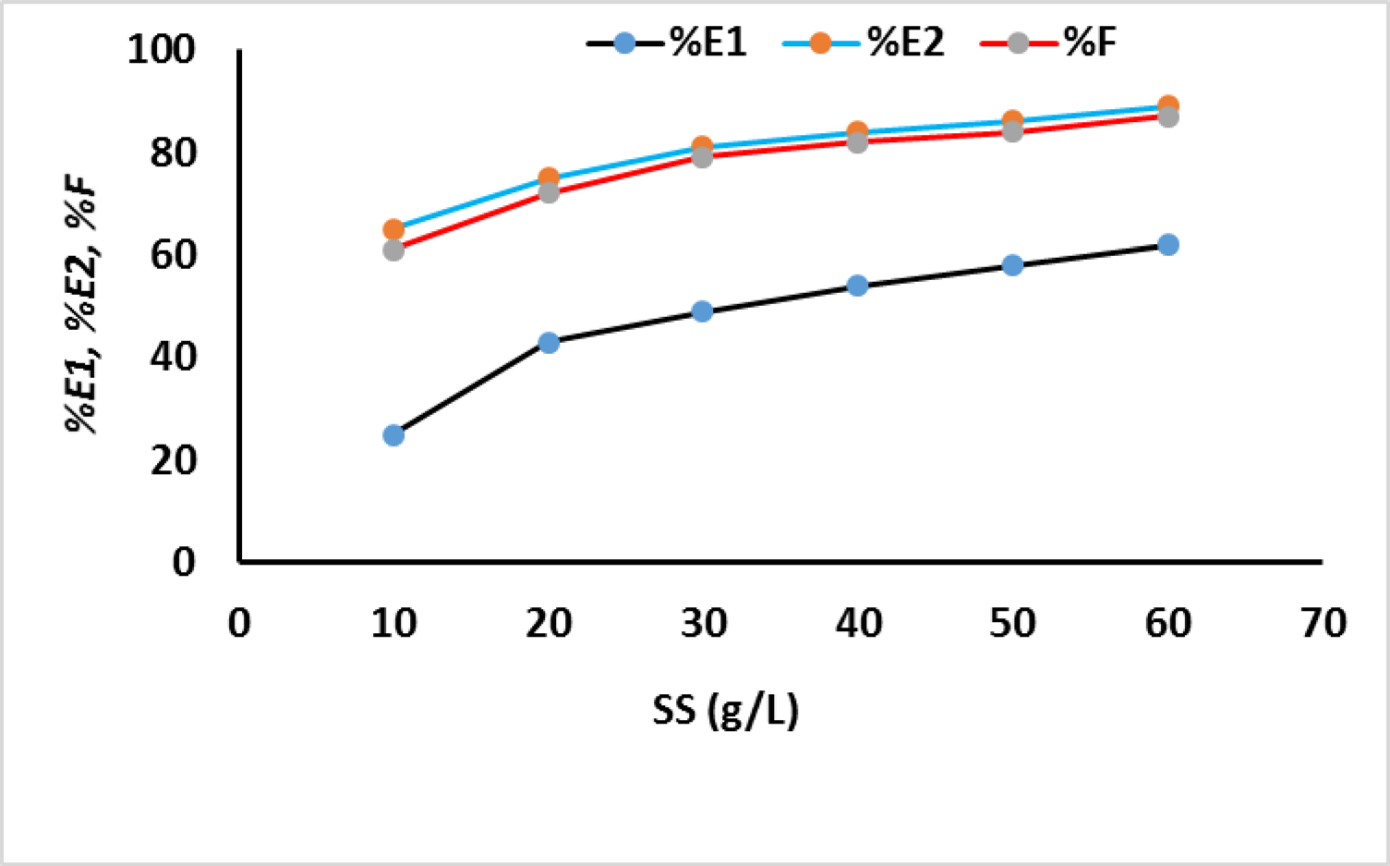
Effect of sod. sulphate concentrations on the dyeing exhaustion and fixation (% *E1* primary exhaustion, % *E2* secondary exhaustion and % *F* the fixation) for the dyed sample 2% owf.

**Fig. 4:**
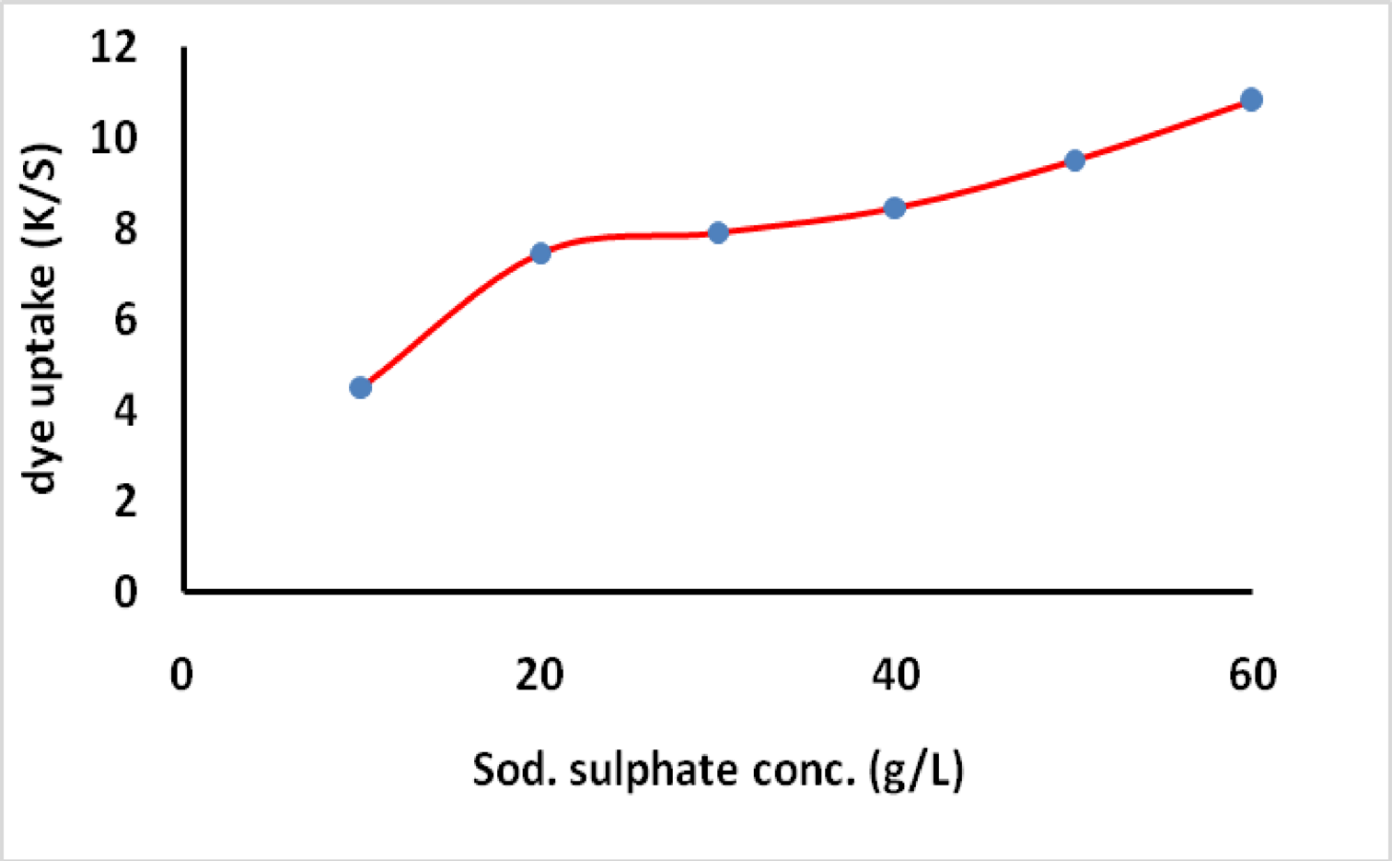
Effect of sod. sulphate concentrations on dye uptake (K/S).

To study the effect of concentration of salt (sod. Sulphate) on the exhaustion and fixation value on cotton fabrics which were dyed with MCT-SES dye, the dyeing process was carried out using different concentration of salt (10–60 g/L) and the exhaustion, fixation was calculated. The dyeing was continue for 60 min, at 60 °C, 2% (owf), L.R (40:1) as presented in Fig. 3, which showing the increase of the dye primary exhaustion (*E1*). The secondary exhaustion *E2* and dye fixation on cotton by increasing salt concentration as presented in Fig. 3. Additionally the dye uptake (k/S) was increase by increase the sod. Sulphate concentration as shown in Fig. 4. This could be due to the relatively small molecular size and lack of substantively conferring moieties. The increasing salt concentration increase the blocking the –ve charge on the surface of the cellulosic fiber which formed due to the ionization of primary hydroxyl group located on the cotton structure which occupied by sodium cation, thus the increase of dye exhaustion and fixation increase the electrostatic interaction between anionic moiety on the dye structure with the introduced + ve site on the cotton fabrics.

#### Effect of alkali concentration

**Fig. 5:**
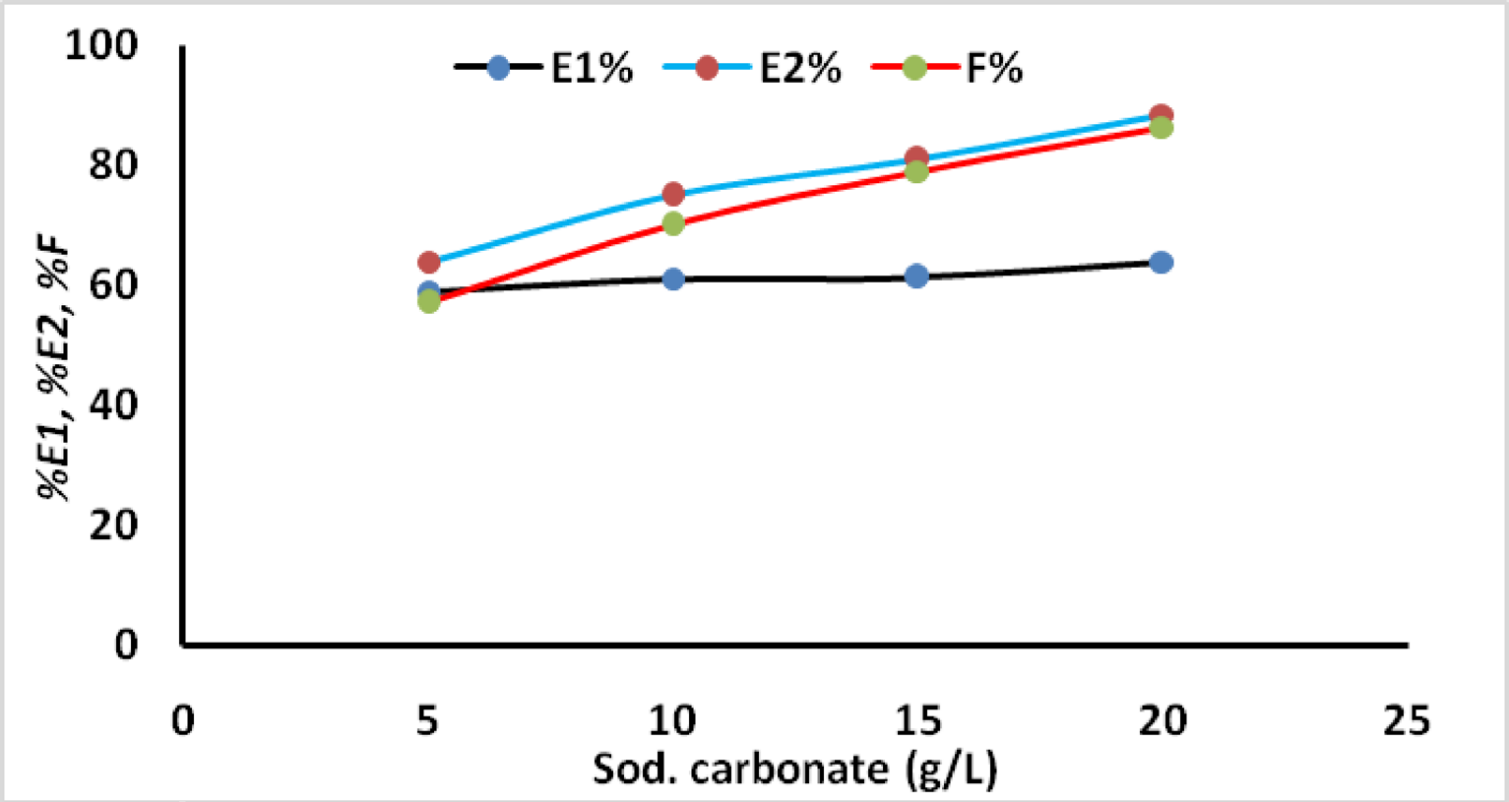
Exhaustion and total fixation yield on dyed cotton at different concentrations of alkali using 2% owf.

**Fig. 6:**
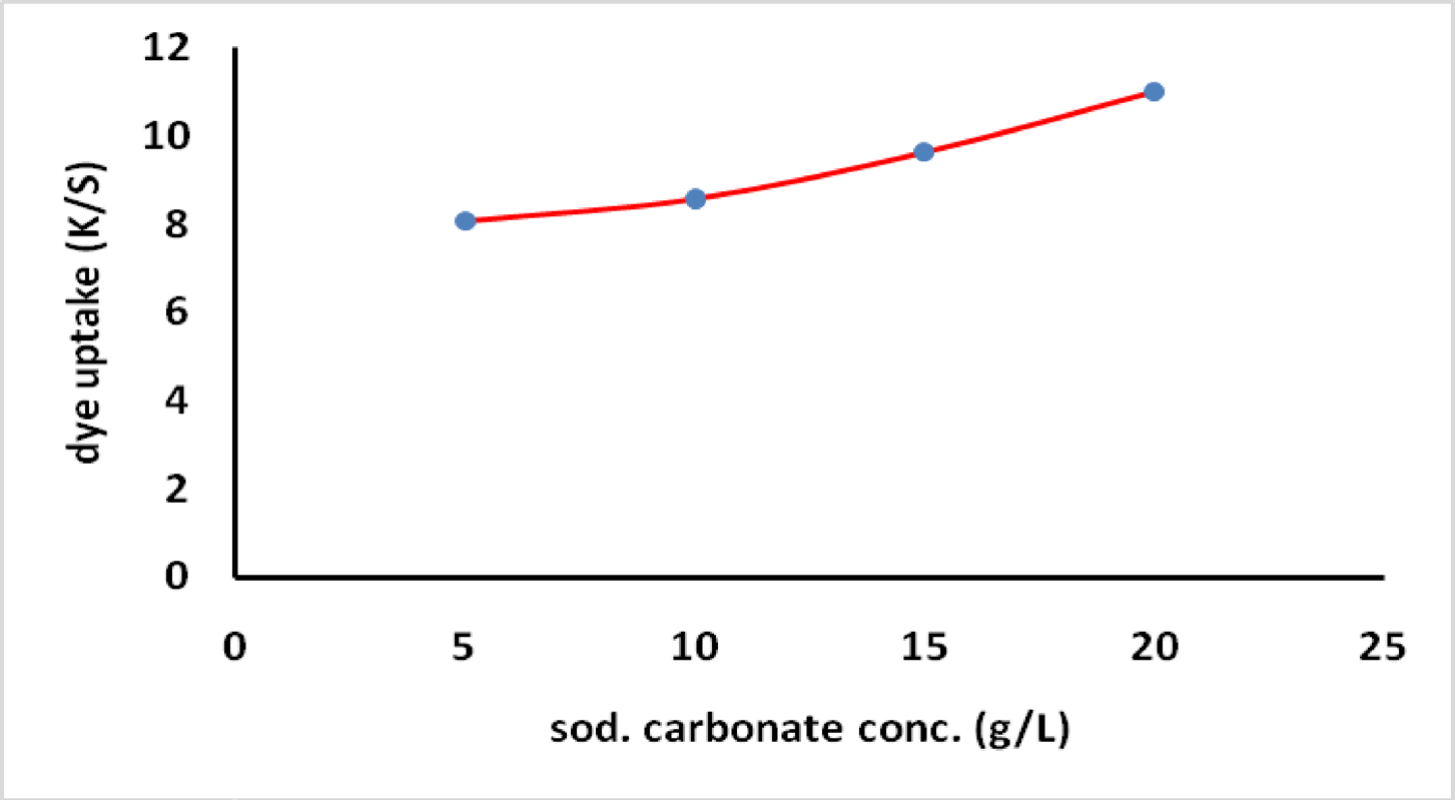
Effect of alkali concentrations on dye uptake.

The effect of sodium carbonate concentration on the dyeing performance of the prepared dye was investigated using various alkali concentrations (5, 10, 15, 20 g/L). The dye uptake for MCT-SES dye through exhaustion and fixation was investigated to assess the influence of alkali concentration on cotton fabric dyeing. The dyeing process lasted for 60 min at 60 °C with a concentration of 2% (owf) and a liquor ratio of 40:1. The dye exhaustion (*%E2*) and dye fixation *(%F*) notably increased with higher sodium carbonate concentrations, as depicted in Fig. 5. The color strength also improved with increasing alkali concentration, as shown in Fig. 6. This enhancement is attributed to the alkali’s role in promoting the formation of covalent bonds between the dye and the fiber. The addition of alkali increases the likelihood of dye-fiber covalent bonding by facilitating the *β*-elimination reaction to convert SES to VS, which then reacts through an addition reaction with the primary hydroxyl group on cotton, forming a covalent bond between the dye and the fiber.

#### Effect of dye concentration

**Fig. 7:**
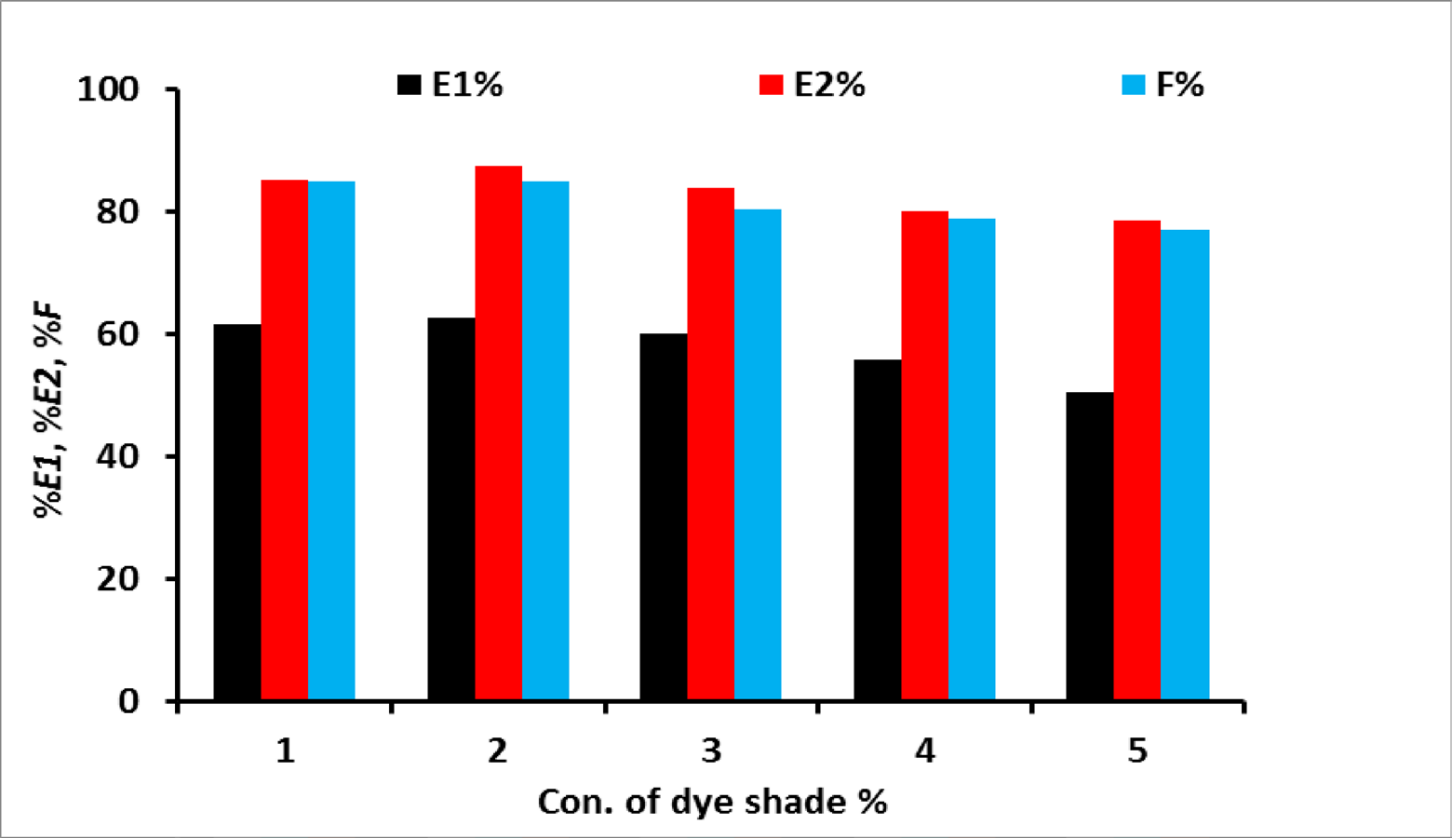
Effect of dye concentrations on dye exhaustion and fixation using 60 g/L sod. sulphate and 20 g/L sod. Carbonate.

**Fig. 8:**
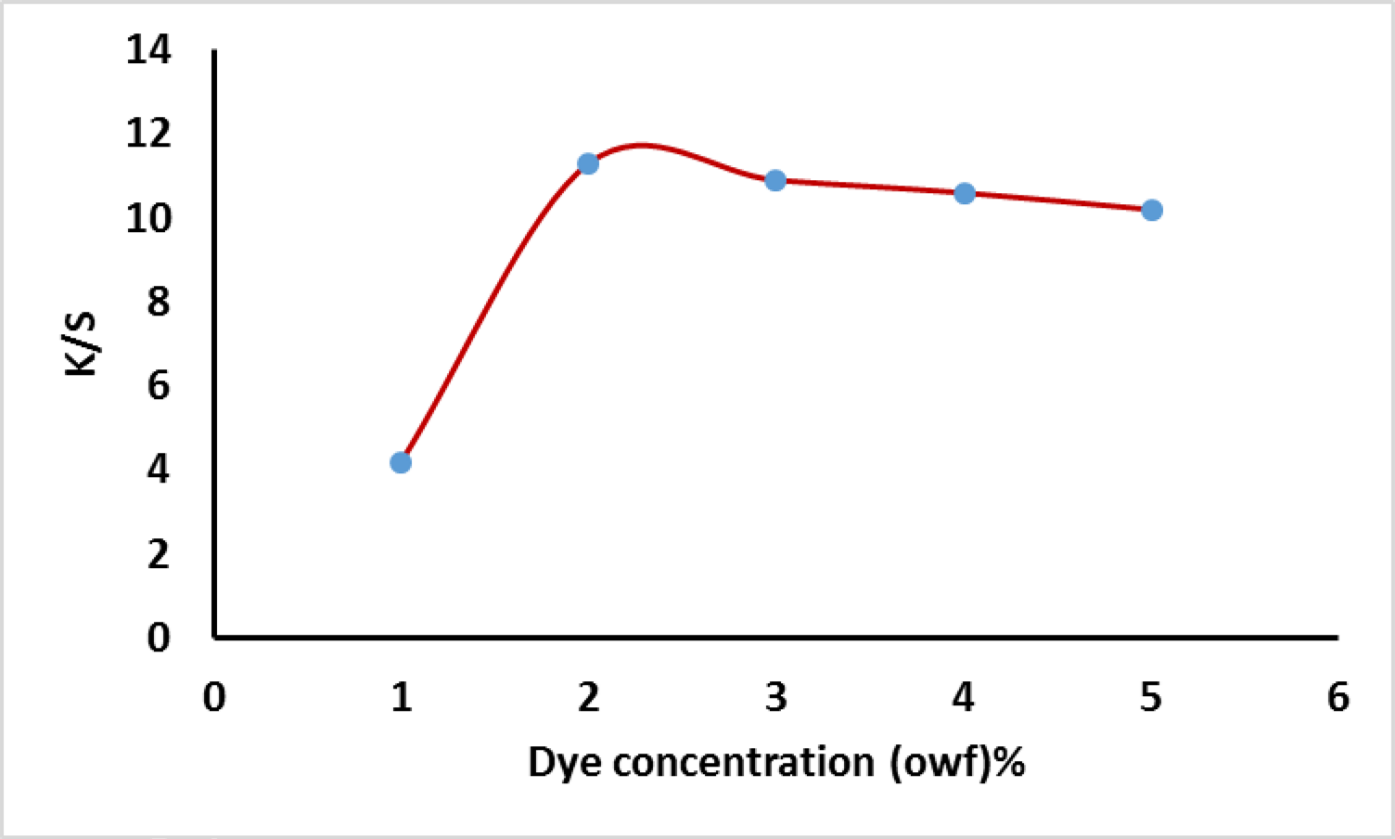
Effect of dye concentration on the K/S using using 60 g/L sod. sulphate and 20 g/L sod. Carbonate.

The application of the prepared dye on cotton for dyeing was presented in Fig. 7 using various concentrations. The dyeing process continued for 60 min at 100 °C with a concentration of 2% (owf) and a liquor ratio of 40:1. The results indicated that lower dye concentrations resulted in higher exhaustion and fixation compared to higher concentrations, as shown in Fig. 7. This is likely due to the increased dye aggregation at higher concentrations, which hinders dye penetration into the fiber. Moreover, higher dye concentrations reduce the number of available dye sites on the fiber, leading to lower exhaustion and fixation yields on cotton fabric. The same effect was observed in the K/S value by increasing dye concentration as presented in Fig. 8.

### Dyeing of wool fabrics

#### Effect of pH

**Fig. 9:**
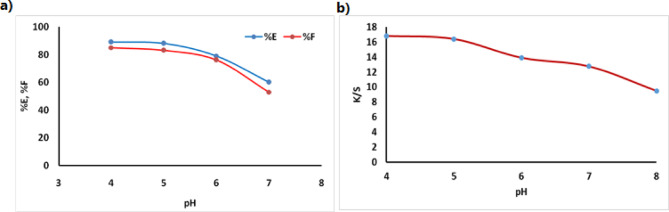
(**a**) Exhaustion and fixation yield of dye on wool at different pH using 2% owf dye shade and (**b**) effect of the pH on k/s value.

The wool fabric was dyed using the prepared dye, showing excellent exhaustion and fixation in acidic conditions as shown in Fig. 9. The dyeing process was carried out at 100 °C for 60 min with 2% (owf) dye concentration and a liquor ratio of 50:1 at different pH levels (4–7). The results in Fig. 9(a) indicate that dye exhaustion and fixation were significantly higher in acidic conditions (pH 4–5) compared to neutral conditions (pH 6–7) due to potential hydrolysis of the dye and interaction of the reactive sites with water instead of the wool fabric. While the same effect was observed in the decrease the K/S value by increasing the pH Fig. 9(b).

#### Effect of dye concentration

**Fig. 10:**
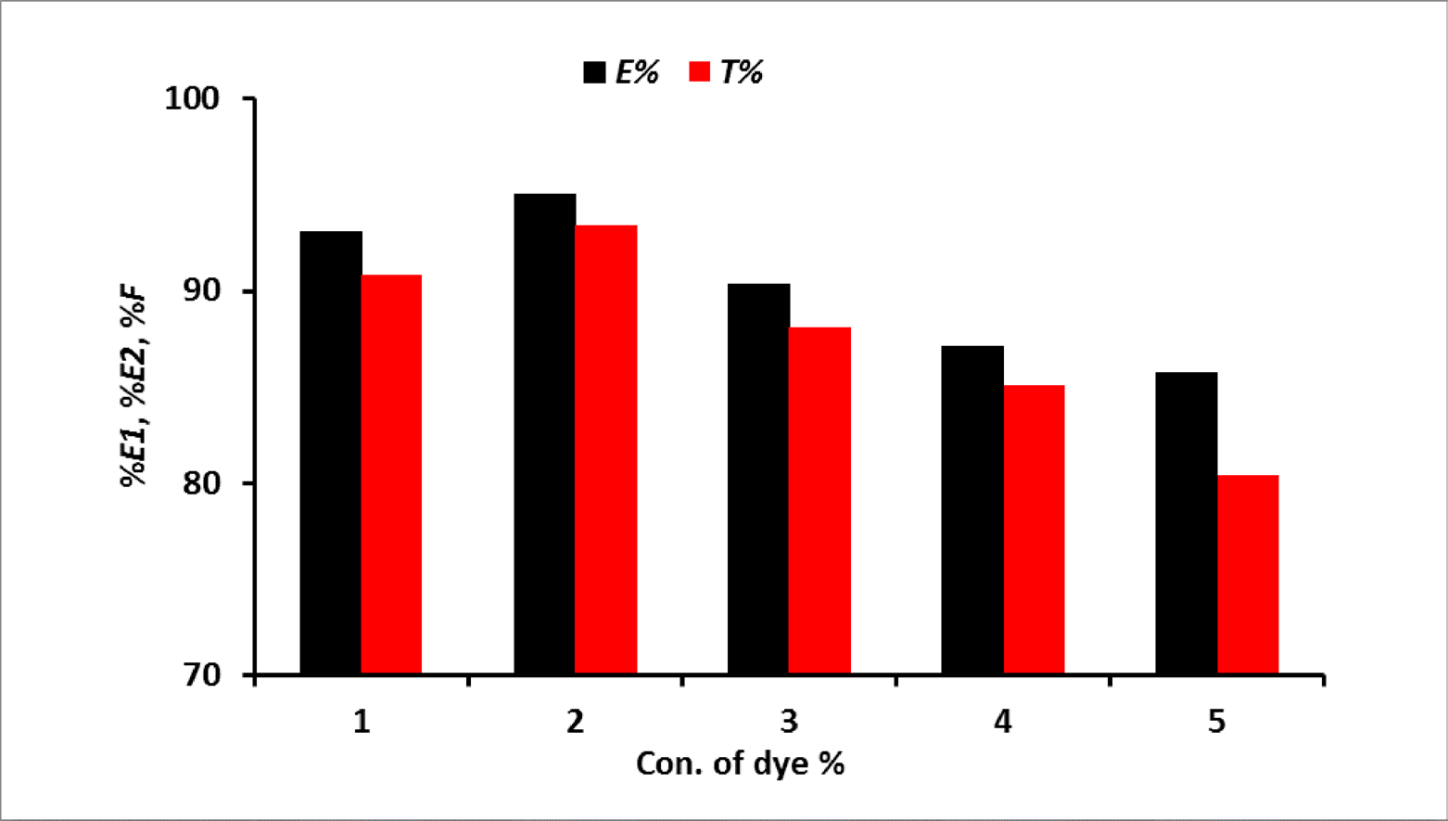
Exhaustion and total fixation yield of dye on wool at different concentration of dye (owf) % at pH5.

**Fig. 11:**
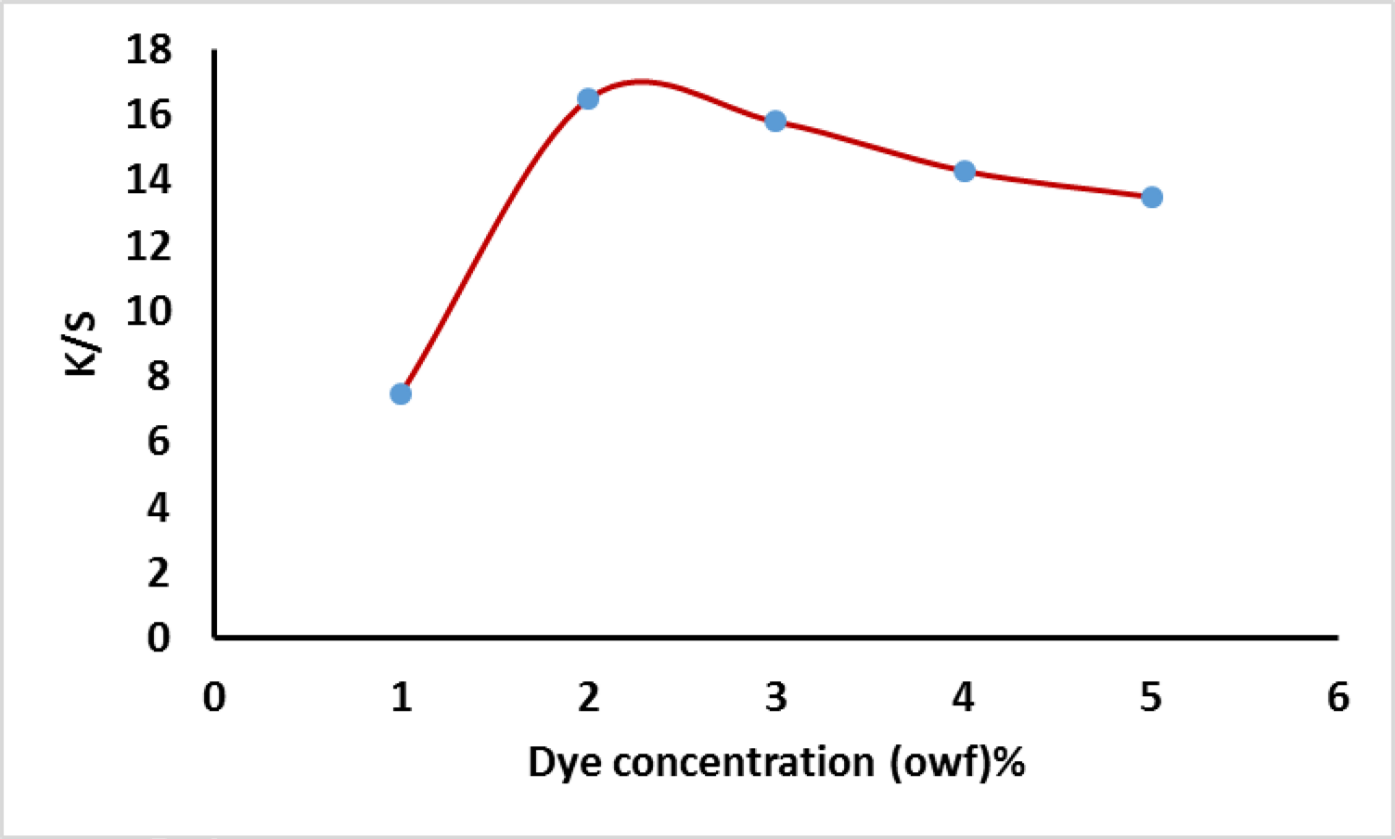
Effect of dye concentration on the K/S using at pH5.

The application of the prepared dye on wool for dyeing was showed in Fig. 10. The dyeing process lasted for 60 min at 100 °C with a liquor ratio of 50:1 and pH 5 for wool. The dye exhaustion and fixation were evaluated at various dye concentrations ranging from 0.5% to 5% owf. It is hypothesized that higher dye concentrations may result in a decrease in the number of available dye sites on the fiber. Additionally, the large molecular size of reactive dyes could hinder exhaustion and fixation efficiency due to steric hindrance and/or electrostatic repulsion, as fixed dye molecules may prevent the approach of additional dye molecules. The effect of the dye concentration on the K/S value was summarized in Fig. 11.

### Fastness properties

The dye fastness on both cotton and wool fabrics were investigated and summarized in Table 1. The dye exhibited excellent fastness properties such as rubbing, washing, perspiration and good light fastness. The high fastness properties of the prepared dye on both cotton and wool fabric ascribed to the chemical interaction of both reactive site (MCT, SES) on cotton and wool as expected forming a strong covalent bond between fabrics and the dye molecule.

**Table 1:** The fastness properties of dyed cotton and wool (2% owf) using MCT-SES dye.

Fabric	Rubbing		Washing*			Perspiration *						light
						Acid			Alkaline			
	Dry	Wet	A	SC	SW	A	SC	SW	A	SC	SW	
Cotton	4–5	4–5	4–5	4–5	4–5	4–5	4–5	4–5	4–5	4–5	4–5	5
Wool	4–5	4–5	4–5	4–5	4–5	4–5	4–5	4–5	4–5	4–5	4–5	4–5

^*^ A = Color change SC = staining on cotton SW = staining on wool.

### Corrosion inhibition

#### Potentiodynamic polarization (PDP) studies

**Fig. 12:**
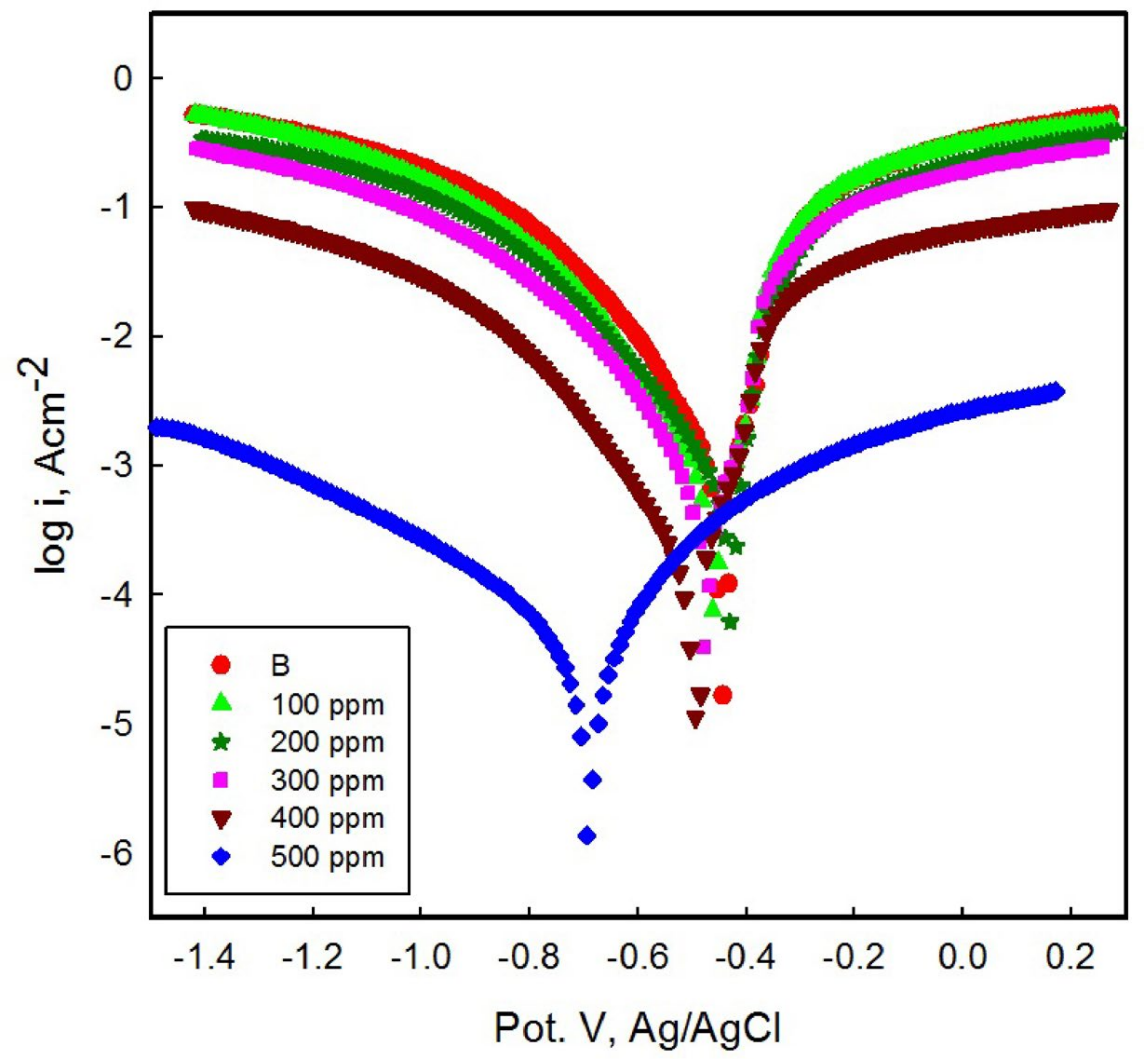
Polarization curves of CS in 1 M HCl without and with different concentrations of the MCT-SES dye.

The polarization curves in Fig. 12 display the carbon steel dissolution in 1 M HCl containing various concentrations of the MCT-SES dye (100–500 ppm). Table 2 summarizes the polarization parameters, such as corrosion resistance (Rpol), corrosion rate (CR), inhibition efficiency (IE %), corrosion potential (*E*_corr_), corrosion current (i_corr_), and the anodic and cathodic Tafel slopes (β_a_ and β_c_).

In the absence of MCT-SES dye, the carbon steel in 1 M HCl exhibited a high corrosion current density of 3.88 × 10^−4^ A. cm^−2^, with anodic and cathodic Tafel slopes (*β*_a_ and *β*_c_) of 0.088 V dec⁻¹ and 0.057 V dec⁻¹, respectively. These values indicate an accelerated rate of iron dissolution and an active hydrogen evolution reaction^22,41^. The low polarization resistance (R_p_ = 38.7 Ω cm²**)** and elevated corrosion rate **(**4.520 mm year⁻¹**)** further emphasize the aggressive nature of the acidic environment^42^. The addition of the MCT-SES dye inhibitor resulted in a noticeable decrease in corrosion current, along with an increase in both Rp and IE%. At a concentration 100 ppm, the I_corr_ decreased to 1.81 × 10⁻⁴ A cm^- 2^, corresponding to an IE of 53.6%. As the concentration increased, the inhibition effect improved gradually, with lower I_corr_ values of 7.76 × 10⁻⁵ A cm^- 2^ at 200 ppm, 6.82 × 10⁻⁵ A cm^- 2^ at 300 ppm, and 1.38 × 10⁻⁵ A cm^- 2^ at 400 ppm, achieving IE% values of 80.15%, 82.47%, and 96.44%, respectively. At the highest concentration (500 ppm), the minimum I_corr_ of 5.29 × 10^−6^ A cm^- 2^ was recorded^22,43^, corresponding to inhibition efficiency of 98.64%, and an increase in R_p_ to 25,430 Ω cm².

These results confirm the excellent protective performance of the MCT-SES dye. Interestingly, the Tafel slopes increased significantly with concentration, with *βa* reaching 0.288 V dec⁻¹ and *βc* rising to 0.163 V dec⁻¹ at 500 ppm. This behavior suggests that the MCT-SES dye effectively impedes both anodic iron dissolution and cathodic hydrogen evolution, through adsorption and the formation of a protective barrier on the CS surface. Additionally, the progressive negative shift in E_corr_ from − 0.443 V to −0.706 V (vs. Ag/AgCl) with increasing MCT-SES dye concentration supports a predominantly cathodic inhibitor behavior^18^.

The MCT-SES dye adsorbs on the metal surface through coordination between lone pairs on heteroatoms within the dye molecule and the unoccupied d-orbitals of iron atoms, as well as interaction between the π-electrons of the aromatic rings and the vacant d-orbitals of the metal atoms. In this process, the Fe atoms’d-orbitals accept electrons from these active sites, facilitating stable adsorption^44,45^.

**Table 2:** Polarization parameters values for CS in 1 M HCl without and with different concentrations of the MCT-SES dye.

Concentration (ppm)	E_corr_ V(Ag/AgCl)	β_a_ (Vdec^− 1^)	β_c_ (Vdec-^1^)	I_corr_ (Acm^− 2^)	*R*_*P*_ (Ω cm^2^)	CR	IE (%)
Blank	−0.443	0.088	0.057	3.88 × 10^− 4^	38.7	4.520 ± 0.2	-
100	−0.463	0.054	0.047	1.81 × 10^− 4^	60.1	2.090 ± 0.4	53.6 ± 1.5
200	−0.491	0.087	0.053	7.76 × 10^− 5^	183.3	0.900 ± 0.3	80.15 ± 1.4
300	−0.504	0.063	0.067	6.82 × 10^− 5^	206.4	0.790 ± 0.5	82.47 ± 1.8
400	−0.688	0.155	0.117	1.38 × 10^− 5^	2098.9	0.160 ± 0.3	96.44 ± 1.1
500	−0.706	0.288	0.163	5.29 × 10^− 6^	25,430	0.0019 ± 0.05	98.64 ± 0.9

**Fig. 13:**
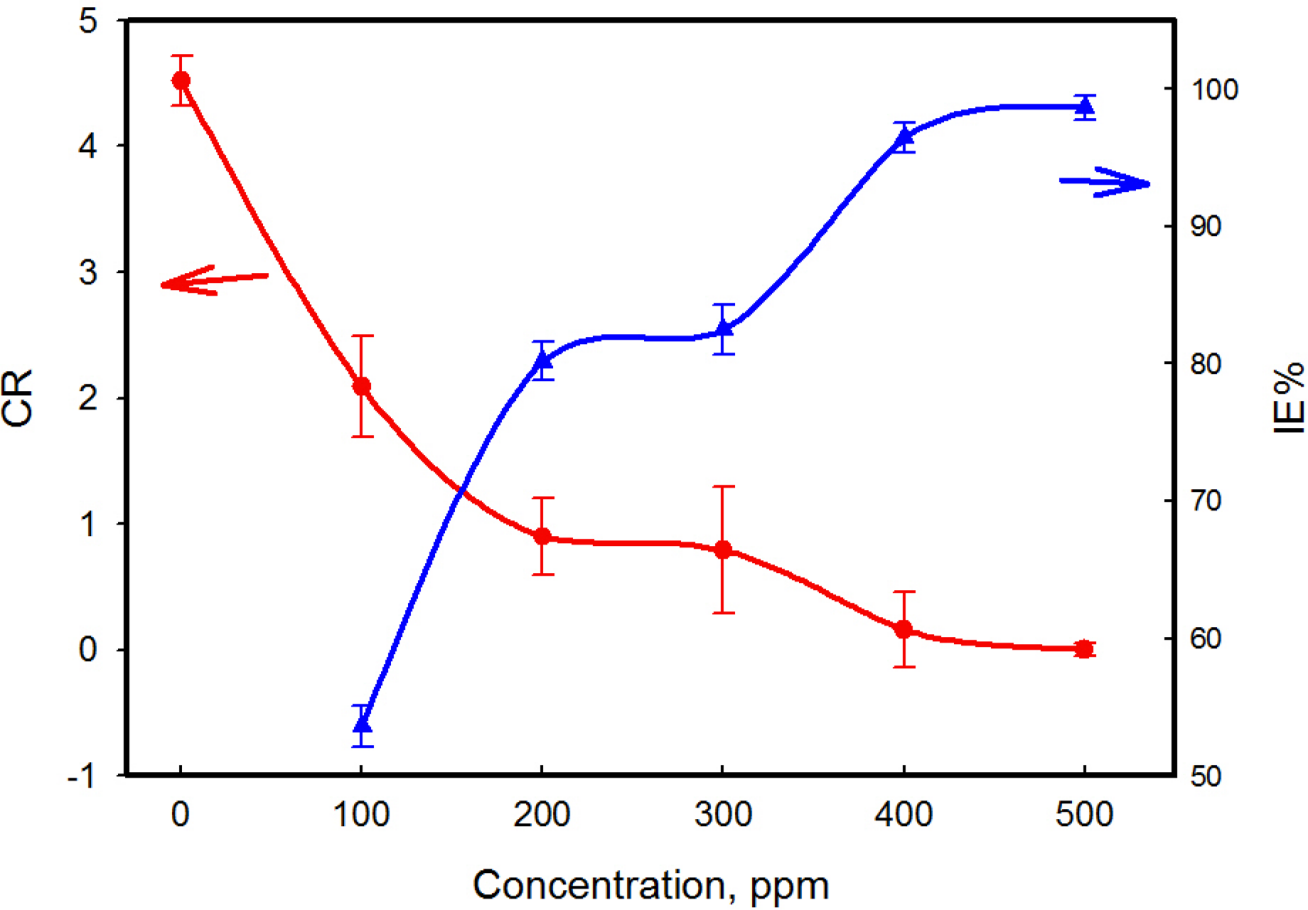
Relation between CR and IE% with different concentration of MCT-SES dye.

It can be observed that the corrosion rate is decreases and the inhibition efficiency (IE %) increases with increasing MCT-SES dye concentration. The highest inhibition efficiency, reaching 98.64% at 500 ppm of the dye, confirms the excellent inhibitor, as shown in Fig. 13, and Table 2.

#### Electrochemical impedance spectroscopy (EIS)

**Fig. 14:**
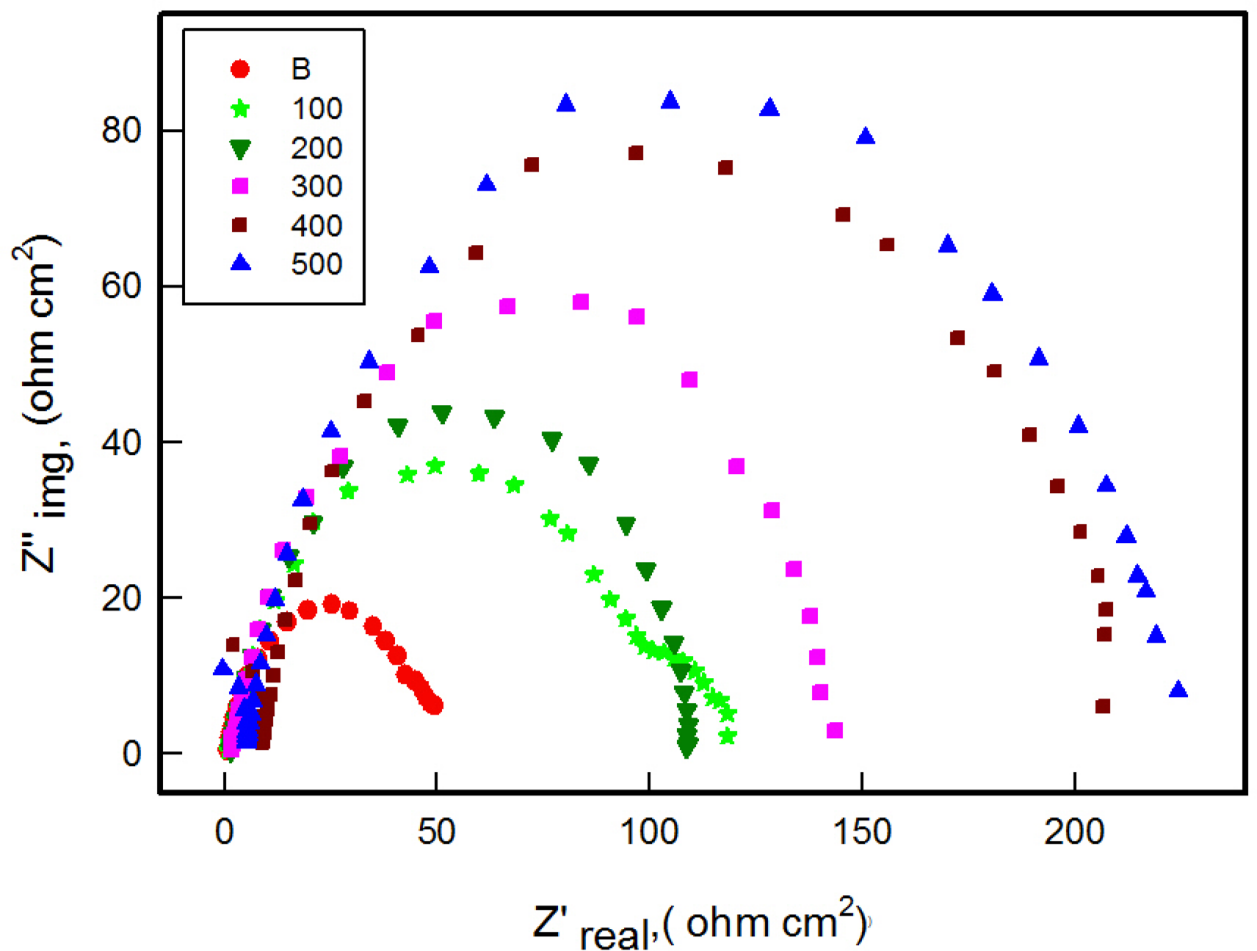
Nyquist plots for carbon steel in 1 M HCl in absence and presence of different concentrations of MCT-SES dye.

Electrochemical impedance spectroscopy was used to further explain the corrosion inhibition performance of the MCT-SES dye on carbon steel immersed in 1 M HCl. As shown in Fig. 14, the Nyquist plots, both in absence and presence of the MCT-SES dye inhibitor, exhibit depressed semicircular shapes, indicating that the corrosion process is charge transfer resistance.

In the blank solution, the Nyquist plot revealed a small semicircle with low impedance, reflecting poor protective characteristics and fast electron transfer at the metal solution interface. With increasing concentrations of MCT-SES dye, the diameters of the semicircles expands, signifying enhanced surface protection and a notable increase in charge transfer resistance^46^. This is attributed to the formation of an adsorbed inhibitor film that acts as an effective physical barrier against corrosion.

**Fig. 15:**
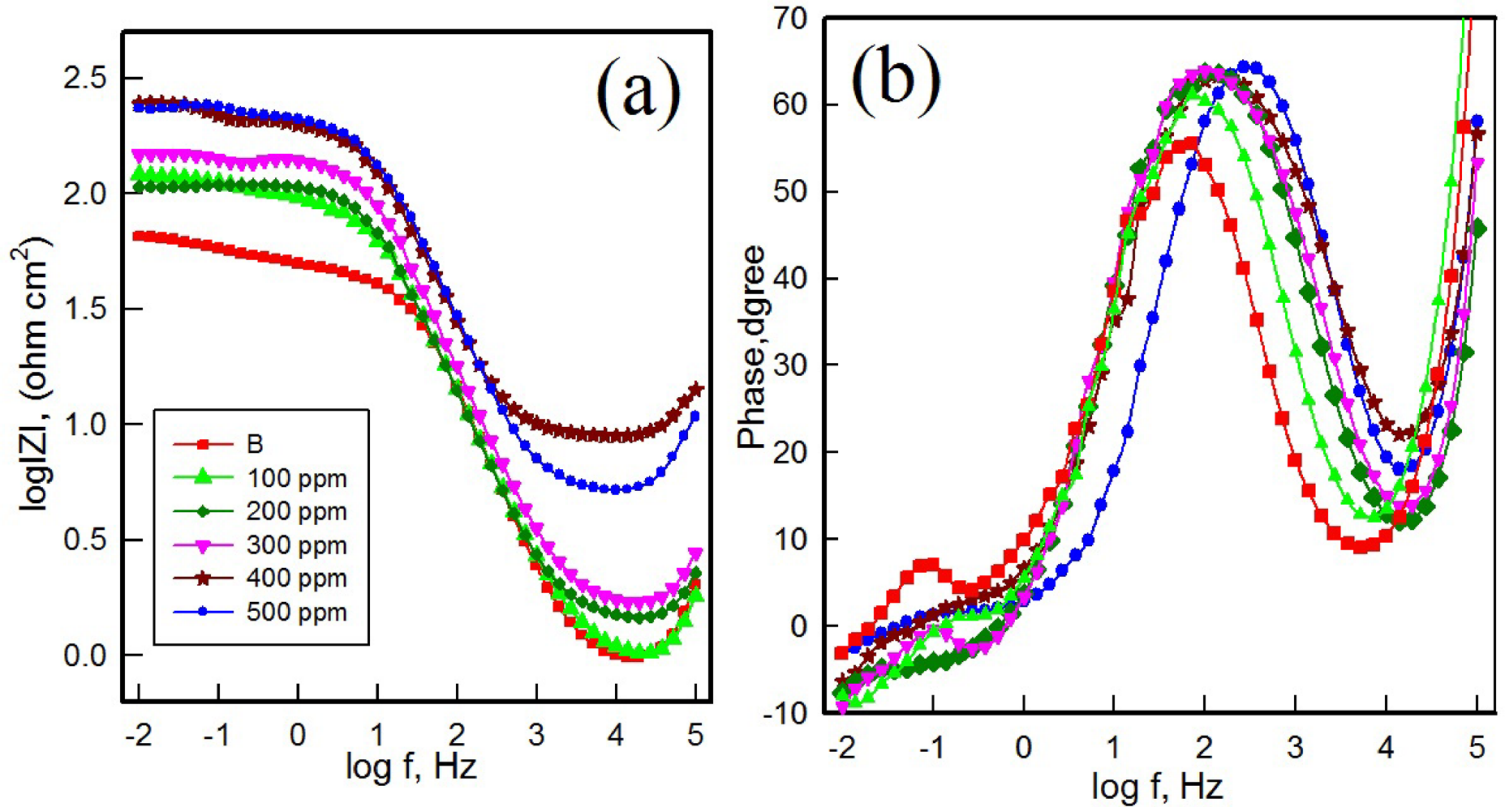
Bode (log f vs. IZI) (**a**) plot and phase angle (log f vs. θ) (**b**) plot of impedance spectra for carbon steel in 1 MHCl in absence and presence of different concentrations of MCT-SES dye.

The Bode magnitude and phase angle plots, shown in Fig. 15(a, b), were analyzed to gain a more understanding of the frequency- dependent electrochemical response of CS in 1 M HCl, both in the absence and the presence of the MCT-SES dye. In the uninhibited solution, the Bode magnitude plot revealed low impedance values across the frequency range, indicating insufficient surface protection. Also, the phase angle plot displayed a low maximum value, further conforming weak barrier^19^. However, in presence of the MCT-SES dye, both the impedance magnitude at low frequencies and the peak phase angle exhibited a progressive increase with increasing MCT-SES dye concentration. At 500 ppm the impedance modulus in the low-frequency region rose by more two orders of magnitude, while the maximum phase angle shifted to approximately to 65 ^o^, indicating improved capacitive behavior and the formation of a more effective protective film on the CS surface. A sharper and more defined peak in the phase plot, especially in the intermediate frequency range, is associated with a well formed, adherent protective film^47,48^.

Overall, the observed changes in the Bode and phase plot responses with increasing MCT-SES dye concentration are consistent with the results in the Nyquist and PDP results, confirming that the MCT-SES dye effectively forms a stable, adsorbed protective film that enhances the corrosion resistance of CS in acidic media.

### Surface morphology (SEM &EDX)

**Fig. 16:**
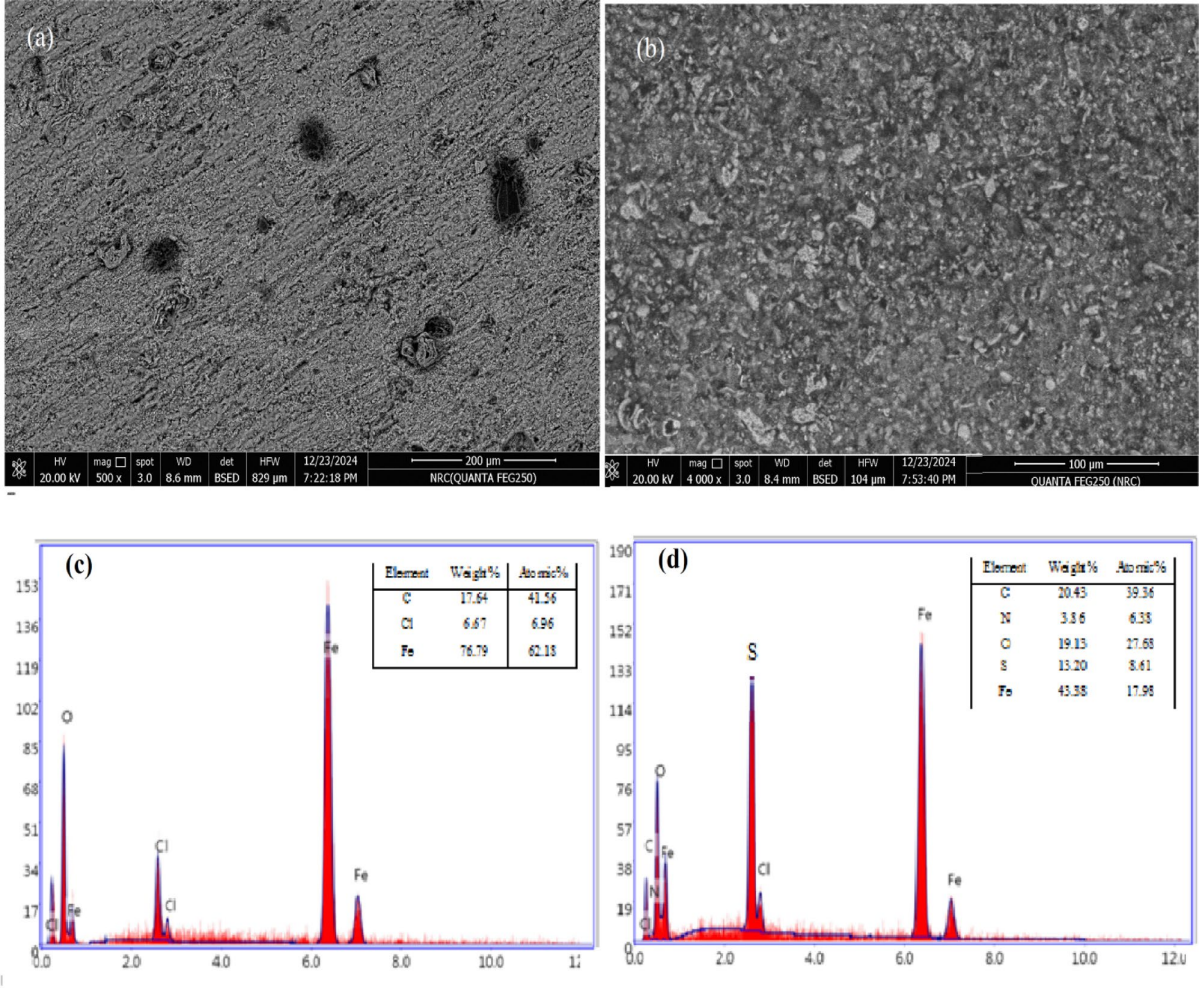
Carbon steel SEM photographs and EDX respectively in 1 M HCl solution without (**a, c**) and (**b, d**) with different concentrations of MCT-SES dye.

Figure 16 presents the SEM images of carbon steel specimens without and with 500 ppm of MCT-SES dye. The uninhibited surface shows severe corrosion damage, evident from its rough texture and pits^49^. In contrast, when using a 500 ppm of MCT-SES dye, the surface shows less damage and smooth, suggesting effective corrosion protection.

Analysis of the uninhibited sample using EDX revealed the presence of iron, chloride, and oxygen, confirming the formation of corrosion products on the carbon steel surface. In contrast, the sample treated with 500 ppm of MCT-SES dye exhibited additional peaks corresponding to sulfur, nitrogen, and carbon. These morphological and composition findings support the formation of a protective film of the MCT-SES dye on the carbon steel surface^50^. These observations align with the inhibition observed in PDP and EIS measurements, confirming the inhibition efficiency of MCT-SES dye.

The remarkable corrosion inhibition efficiency of synthesized isatin-based MCT-SES dye is strongly attributed to its molecular structure. The heteroatoms (N, O, and S) and function groups as (C = O), (NH2), and (SO3H) together with π conjugation framework, facilitate effective adsorption on CS surface. This adsorbed film hinders both anodic dissolution of iron and cathodic hydrogen evolution. Additionally, the presence of monochlorotriazine and sulphatoethylsuphone improve the adsorption behavior, resulting protective film that impede the diffusion of aggressive ions to the metal surface.

Therefore, the higher inhibition efficiency and stability of MCT-SES dye in HCl solution can be attributed to its molecular structure and electron donating groups that enhance its adsorption on the CS surface. The inhibition efficiency of the synthesized MCT-SES dye was compared with previously reported derivatives, as summarized in Table 3. G exhibited higher performance, achieving over 98.64% efficiency at 500 ppm. These findings underscore the potential of dye as an efficient, eco-frindly, and cost effective corrosion inhibitor for carbon steel in 1 M HCl.

**Table 3:** Comparison of Inhibition efficiency (IE %) of the synthesized --- with other reported studies.

Inhibitor	IE%	Conc.	Reference
isatin Schiff bases	95	100 ppm	45
3,3′,3′′-((1,3,5-triazine-2,4,6-triyl)tris(azaneylylidene))tris(indolin-2-one	91.51	2 × 10^− 3^ mM	20
− (1-ethoxycarbonylmethyl-2-o xoindolin-3-ylidene) thiosemicarbazide	87%	400 mg/L	51
2‑(2‑oxoindolin‑3‑ylidene) hydrazinecarbothioamide	96.7	0.5 mM	48
Isatin Schiff base (OHMHI	93.4	0.5 mM	50
A Schiff base, 3-(phenylimino)indolin-2-one,	81.9	1 × 10^− 3^ M	52
piperidinylmethylindoline-2-on	91	300 ppm	53
3,3- (1,4-phenylenebis (azan-1-yl-1-ylidene))diindolin-2-one	84	10^− 3^ M	54
2-methoxybenzylidene) hydrazono) indolin-2-one	92.3	250 ppm	55
Salycilidene isatin hydrazine sodium sulfonate	87.8	10^− 5^ M	19
N-(Piperidinomethyl)−3-s(pyridylidene)amino]isatin	94	300 ppm	56
monochlorotriazine/sulphatoethylsulphone bearing isatin moiety (MCT-SES dye),	98.64	500 ppm	Recent work

### Antimicrobial evaluation

**Fig. 17:**
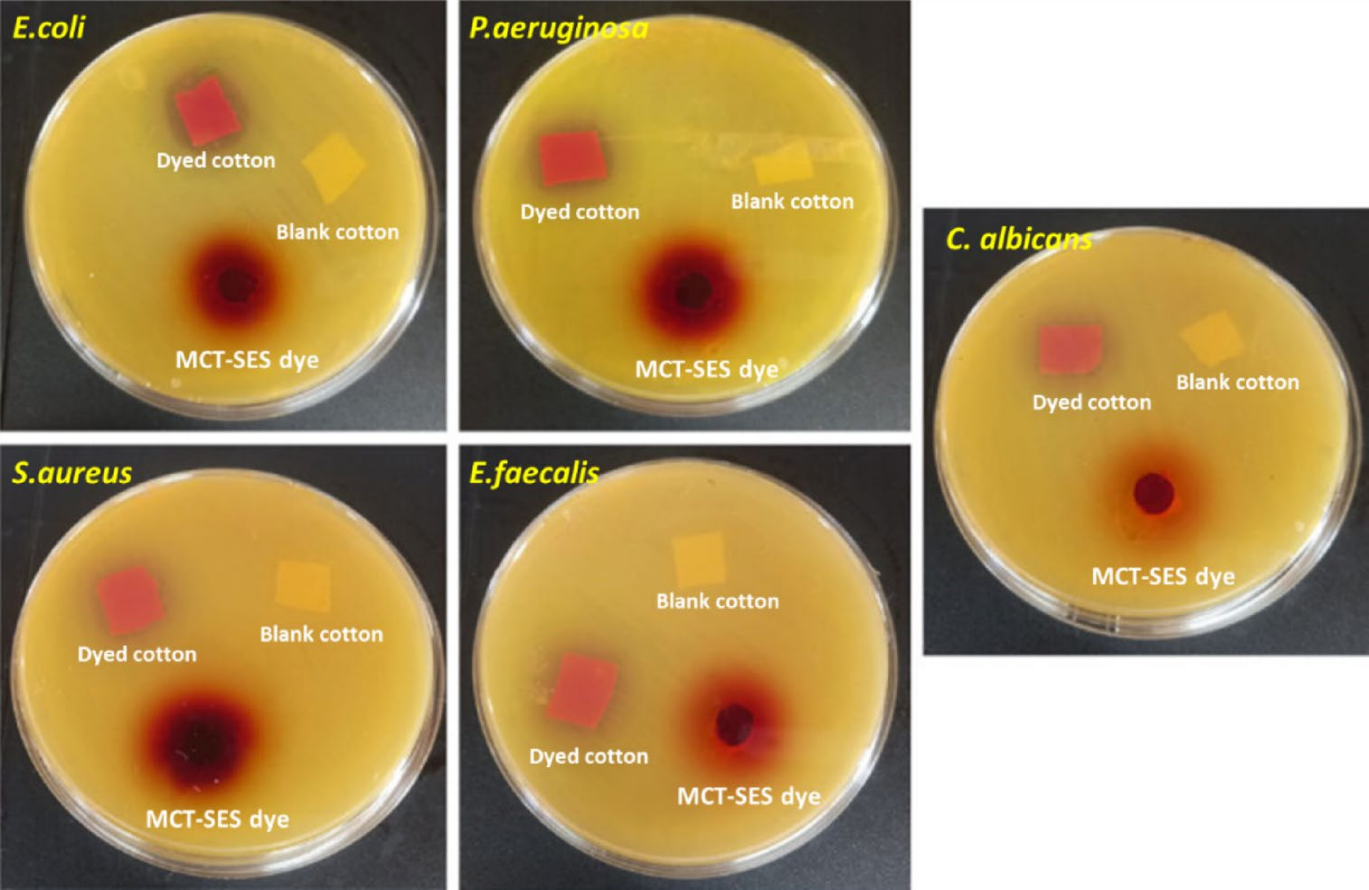
Digital photographic images of Petri-dishes illustrating the DIZ of all materials against some pathogenic microbes.

The antimicrobial activity of blank cotton, dyed cotton, and the antimicrobial dye MCT-SES was evaluated against a panel of pathogenic microorganisms, including *E. coli*, *P. aeruginosa*, *S. aureus*, *E. faecalis*, and *C. albicans*, using the diameter of inhibition zones (DIZ) as an indicator. As shown in Table 4; Fig. 17, blank cotton exhibited no antimicrobial effect (0 ± 0.0 mm) against any of the tested microbes, confirming the absence of inherent antimicrobial properties. In contrast, dyed cotton treated with MCT-SES dye displayed notable antimicrobial activity, with inhibition zones ranging from 5 ± 0.52 mm (*E. faecalis*) to 11 ± 0.25 mm (*E. coli*), demonstrating that the antimicrobial dye retained functional efficacy when applied to the cotton substrate.

The MCT-SES dye alone exhibited the highest antimicrobial efficacy, particularly against *P. aeruginosa* (23 ± 0.23 mm) and *E. coli* (20 ± 0.34 mm), indicating a strong broad-spectrum antimicrobial profile. Interestingly, while dyed cotton showed no activity against *C. albicans*, the pure dye still demonstrated a moderate inhibitory effect (14 ± 0.49 mm), suggesting possible limitations in dye fixation or leaching that may influence antifungal performance when bound to the fabric.These findings collectively underscore the successful functionalization of cotton textiles with MCT-SES dye, achieving enhanced antibacterial properties, and highlight the importance of optimizing dye-fiber interactions for broad-spectrum antimicrobial efficacy.

**Table 4:** Antimicrobial potential and measured IZD (mean ± SD) of blank cotton, dyed cotton and MCT-SES dye against some pathogenic microbes.

Selected microbes	Diameters of the inhibition zones (DIZ), mm		
	Blank cotton	Dyed cotton	MCT-SES dye
*E.coli*	0 ± 0.0	11 ± 0.25	20 ± 0.34
*P.aeruginosa*	0 ± 0.0	10 ± 0.43	23 ± 0.23
*S. aureus*	0 ± 0.0	7 ± 0.62	19 ± 0.62
*E. faecalis*	0 ± 0.0	5 ± 0.52	17 ± 0.73
*C. albicans*	0 ± 0.0	0 ± 0.0	14 ± 0.49

**Fig. 18:**
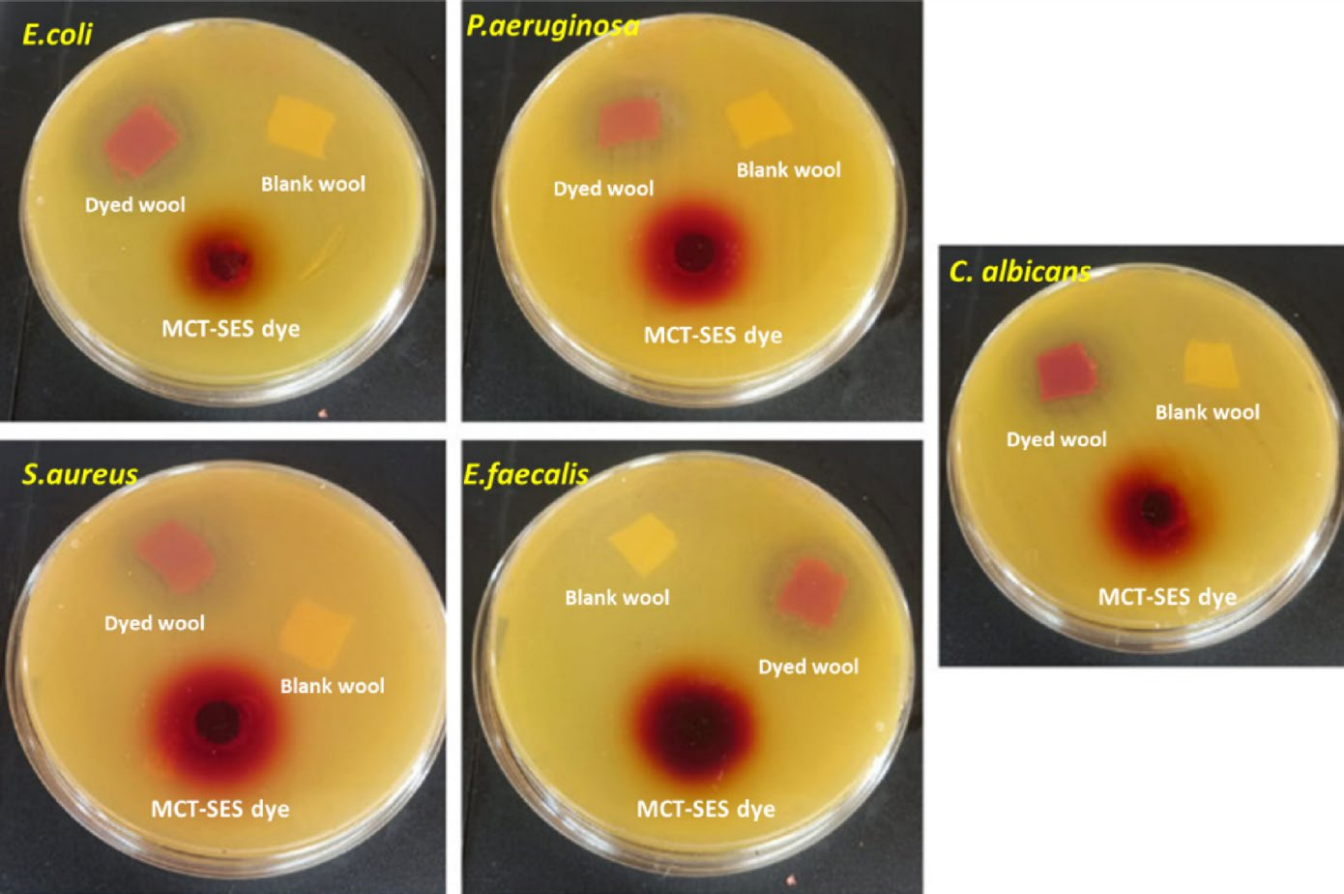
Digital photographic images of Petri-dishes illustrating the DIZ of all materials against some pathogenic microbes.

Table 5; Fig. 18 present the antimicrobial efficacy of blank wool, dyed wool treated with MCT-SES dye, and the pure dye itself against various pathogenic microorganisms. As with cotton, blank wool exhibited no inhibitory activity (0 ± 0.0 mm), affirming its lack of inherent antimicrobial properties. Upon dyeing with MCT-SES dye, the wool substrate displayed significant antimicrobial potential, with inhibition zones ranging from 13 ± 0.71 mm (*C. albicans*) to 21 ± 0.18 mm (*E. coli*), indicating successful incorporation of the bioactive dye and its retention on the wool fibers. Interestingly, dyed wool exhibited even greater activity than the pure dye in the case of *E. coli* (21 ± 0.18 mm vs. 19 ± 0.42 mm), suggesting that the wool matrix may enhance dye stability or facilitate a sustained release effect. In contrast, the MCT-SES dye alone remained more effective against *P. aeruginosa* (23 ± 0.65 mm vs. 19 ± 0.63 mm in dyed wool), possibly due to higher immediate bioavailability in solution. The dyed wool also demonstrated strong inhibitory activity against Gram-positive bacteria such as *S. aureus* (17 ± 0.82 mm) and *E. faecalis* (16 ± 0.54 mm), as well as moderate antifungal efficacy against *C. albicans* (13 ± 0.71 mm), confirming the dye’s broad-spectrum activity. These results highlight the effectiveness of MCT-SES as a potent antimicrobial agent for treating wool fibers, leading to substantial suppression of both Gram-negative and Gram-positive bacteria, along with fungal pathogens. The superior or comparable activity of dyed wool relative to the free dye in several cases highlights the suitability of wool as a compatible and effective substrate for antimicrobial textile applications.

**Table 5:** Antimicrobial potential and measured IZD (mean ± SD) of blank wool, dyed wool and MCT-SES dye against some pathogenic microbes.

Selected microbes	Diameters of the inhibition zones (DIZ), mm		
	Blank wool	Dyed wool	MCT-SES dye
*E.coli*	0 ± 0.0	21 ± 0.18	19 ± 0.42
*P.aeruginosa*	0 ± 0.0	19 ± 0.63	23 ± 0.65
*S. aureus*	0 ± 0.0	17 ± 0.82	20 ± 0.19
*E. faecalis*	0 ± 0.0	16 ± 0.54	19 ± 0.38
*C. albicans*	0 ± 0.0	13 ± 0.71	14 ± 0.52

### The inhibitory effect of dyed cotton and wool fabrics

**Fig. 19:**
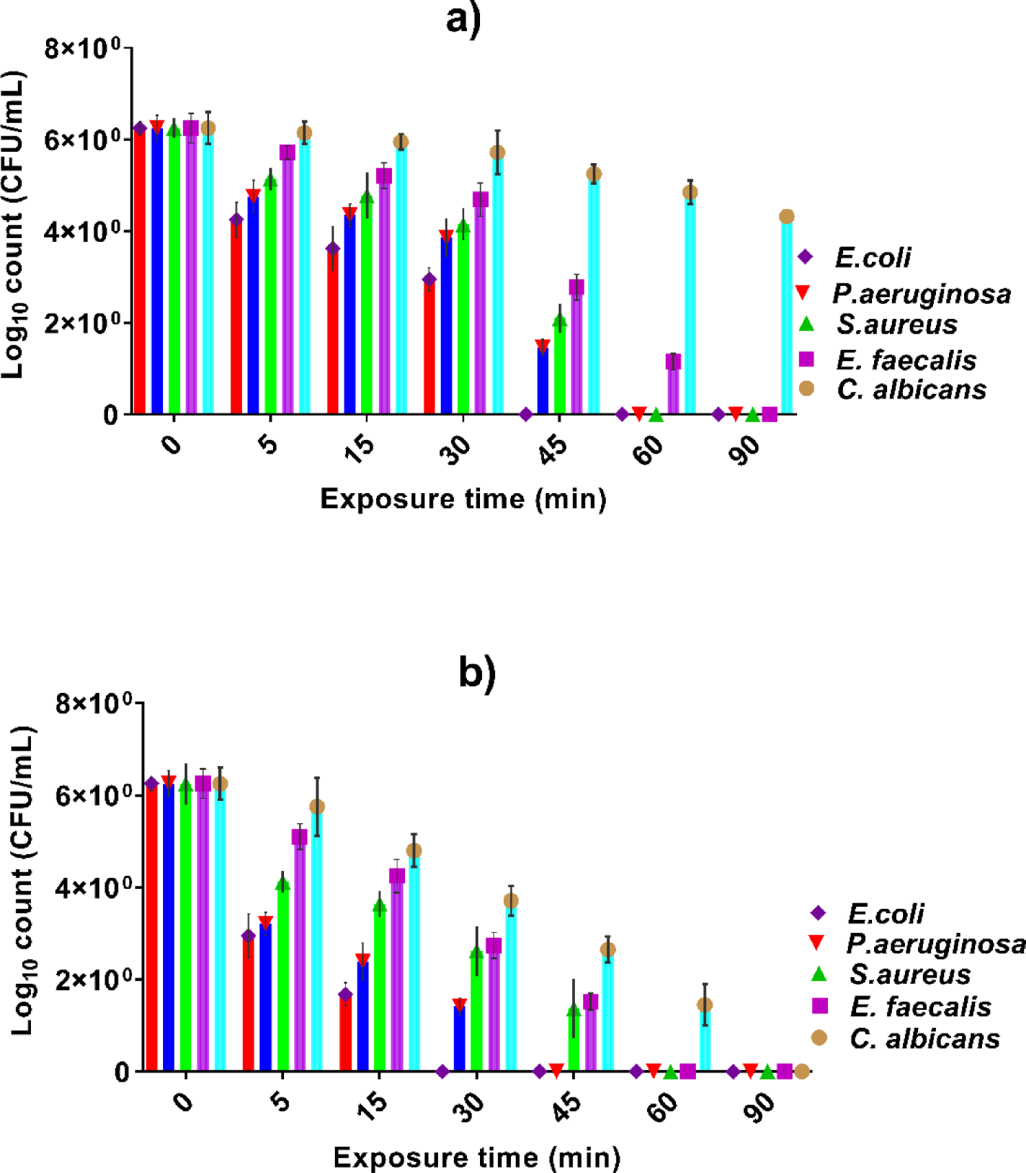
Time-dependent antimicrobial activity of (**a**) MCT-SES-dyed cotton and (**b**) MCT-SES-dyed wool against selected pathogenic microbes, showing reduction in log₁₀ CFU/mL of *E. coli*,* P. aeruginosa*,* S. aureus*,* E. faecalis*, and *C. albicans* over an exposure period of 0 to 90 min.

The antimicrobial time-kill kinetics of dyed cotton treated with MCT-SES dye were evaluated against five pathogenic microorganisms, as illustrated in Fig. 19(a). Initially, all tested microbes, including *E. coli*, *P. aeruginosa*, *S. aureus*, *E. faecalis*, and *C. albicans*, exhibited comparable bacterial and fungal loads of approximately 6.25 log₁₀ CFU/mL at time zero. Upon exposure to the dyed cotton, a significant time-dependent reduction in microbial count was observed. Among the bacteria, *E. coli* and *P. aeruginosa* were the most susceptible, with their populations dropping below detectable levels by 60 min of contact, indicating rapid and complete bactericidal activity. Similarly, *S. aureus* and *E. faecalis* also showed marked reductions, reaching near-complete elimination by 60 min. In contrast, *C. albicans* demonstrated more resistance, showing only a gradual decline in viable count, reaching approximately 4.32 log₁₀ CFU/mL by 90 min. These findings confirm that the dyed cotton exhibits potent and fast-acting antibacterial effects, particularly against both Gram-negative and Gram-positive bacteria, while showing moderate antifungal efficacy. This supports its potential application in antimicrobial textile development for effective infection control. The high affinity of the dye against bacteria may attributed to high interaction of the active sites in dye structure such as isatin amide group and also the ethylsulphone moiety with the bacteria protein.

The antimicrobial time-kill curve of dyed wool functionalized with MCT-SES dye, as depicted in Fig. 19(b), reveals a potent and time-dependent inhibitory effect against a range of pathogenic microorganisms, including *E. coli*, *P. aeruginosa*, *S. aureus*, *E. faecalis*, and *C. albicans*. At the initial time point (0 min), all microbial strains started with similar viable counts (~ 6.25 log₁₀ CFU/mL). A marked reduction in microbial load was observed over time, particularly among the bacterial strains. *E. coli* and *P. aeruginosa* showed rapid susceptibility to the dyed wool, with complete elimination achieved by 45 and 60 min, respectively. *S. aureus* and *E. faecalis* also responded significantly, with bacterial counts dropping below detectable levels by 60 min. Notably, *C. albicans* exhibited a slower decline in CFU/mL count compared to bacteria, maintaining a viable population of ~ 1.45 log₁₀ CFU/mL even at 60 min, but was completely eradicated by 90 min. These findings demonstrate that dyed wool possesses strong bactericidal and fungicidal activity, with slightly delayed antifungal effects relative to antibacterial ones. Overall, the results confirm that MCT-SES-dyed wool is highly effective in reducing microbial viability, supporting its potential application in antimicrobial textiles intended for broad-spectrum pathogen control.

#### SEM examination

**Fig. 20:**
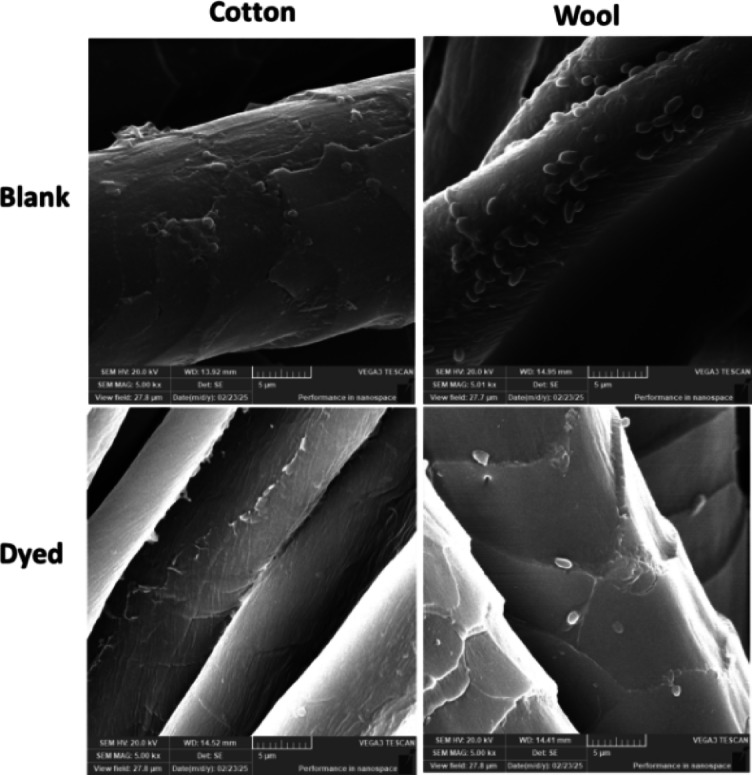
SEM microimages of the surface of cotton and wool fabrics after exposure to *P. aeruginosa*. Top panels: Blank cotton and wool exhibit dense colonization with intact, healthy bacterial cells. Bottom panels: Dyed cotton and wool (treated with MCT-SES dye) show markedly reduced bacterial adherence and visible morphological damage to the bacterial cells, including membrane disruption and cell lysis, indicating strong antimicrobial activity.

Figure 20 presents SEM micrographs illustrating the surface morphology of blank and MCT-SES-dyed cotton and wool fabrics following exposure to *Pseudomonas aeruginosa*. The images provide visual evidence of the antimicrobial impact of the dyed materials. In the upper panels, blank cotton and blank wool surfaces exhibit dense colonization by intact, rod-shaped P. aeruginosa cells, indicative of healthy bacterial proliferation without antimicrobial intervention. These bacterial cells appear structurally intact, with smooth membranes and consistent morphology, confirming that the untreated textiles do not inhibit microbial growth. Conversely, the lower panels corresponding to dyed cotton and dyed wool samples demonstrate a marked reduction in bacterial density. Moreover, the few bacterial cells on the dyed surfaces show visible signs of structural deformation, including membrane rupture, wrinkling, and lysis. This cellular disruption suggests that the MCT-SES dye incorporated into the textile fibers exerts a strong bactericidal effect, likely by compromising the integrity of the bacterial cell envelope. The cleaner surfaces and sparse, morphologically altered bacteria support the quantitative antimicrobial findings and confirm that the treated fabrics effectively inhibit *P. aeruginosa* attachment, survival, and proliferation.

**Fig. 21:**
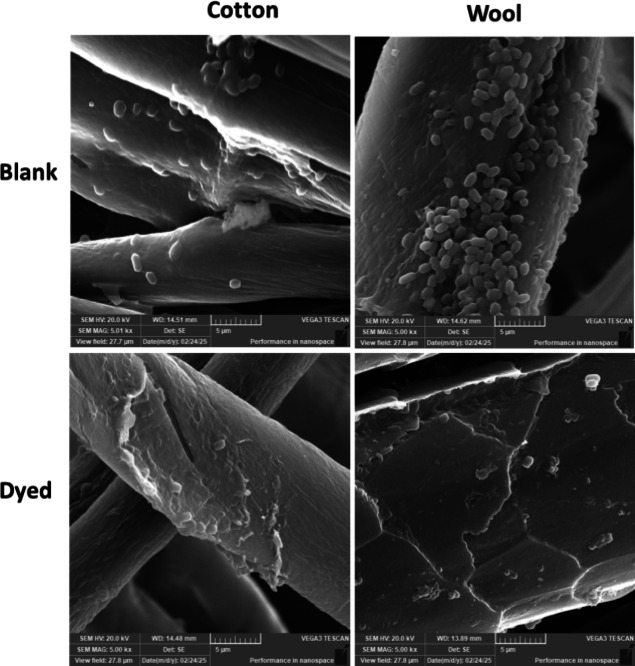
SEM microimages of the surface morphology of cotton and wool fabrics after exposure to *E. faecalis*. Top panels: Blank cotton and wool display heavy colonization with intact bacterial cells adhering to the fiber surfaces, indicating unhindered microbial growth. Bottom panels: dyed cotton and wool treated with MCT-SES dye show a substantial reduction in bacterial attachment, with fewer cells observed and visible membrane deformation and surface disruption, confirming the antibacterial efficacy of the dyed materials.

The SEM micrographs depicted in Fig. 21 provide direct morphological evidence of the antimicrobial action exerted by MCT-SES-dyed cotton and wool fabrics against *Enterococcus faecalis*. In the images corresponding to the blank (untreated) cotton and wool (top panels), a dense population of *E. faecalis* cells is clearly observed adhering to the fiber surfaces. These bacterial cells appear morphologically intact, with smooth, rounded shapes typical of healthy cocci, indicating that the untreated textiles offer no inhibitory effect on bacterial colonization or growth. In contrast, the lower panels, representing the dyed cotton and dyed wool samples, demonstrate a significant reduction in bacterial adhesion. The few remaining *E. faecalis* cells exhibit notable morphological damage, including surface deformation, membrane rupture, and in some areas, complete absence of bacterial presence. These structural alterations are indicative of bacterial cell death likely caused by direct contact with the antimicrobial dye (MCT-SES) embedded in the textile fibers. Overall, these observations are consistent with the antimicrobial assay results and confirm the strong bactericidal activity of the dyed fabrics. The images validate that MCT-SES not only inhibits bacterial proliferation but also compromises bacterial membrane integrity, leading to cell lysis. This highlights the effectiveness of dyed cotton and wool as promising antimicrobial textile materials for controlling *E. faecalis* contamination in hygiene-sensitive applications.

#### In silico molecular docking

Molecular docking analysis was conducted to assess the potential binding affinity and interaction modes of PES-disulphide with key bacterial proteins implicated in cell wall synthesis, membrane permeability, and DNA replication. The selected protein targets included penicillin-binding proteins (PBPs), outer membrane porins (OPR), and DNA gyrase subunits (GyrB) from *Pseudomonas aeruginosa*, *Acinetobacter baumannii*, *Staphylococcus aureus*, and *Streptococcus mutans*, chosen for their essential roles in bacterial viability and their relevance in antibiotic resistance mechanisms.

Table 6 presents comparative 3D and 2D structural visualizations of eight bacterial proteins that are potential drug targets, including proteins from *E*. coli (mdtK and PBPA), Pseudomonas species (PS-ARG-mrcA and PS-ARG-OPR), Staphylococcus aureus (DNA gyrase B and penicillin-binding protein), and Staphylococcus epidermidis (DNA gyrase B and penicillin-binding protein), providing essential structural context for understanding their binding sites and molecular functions.

**Table 6:**
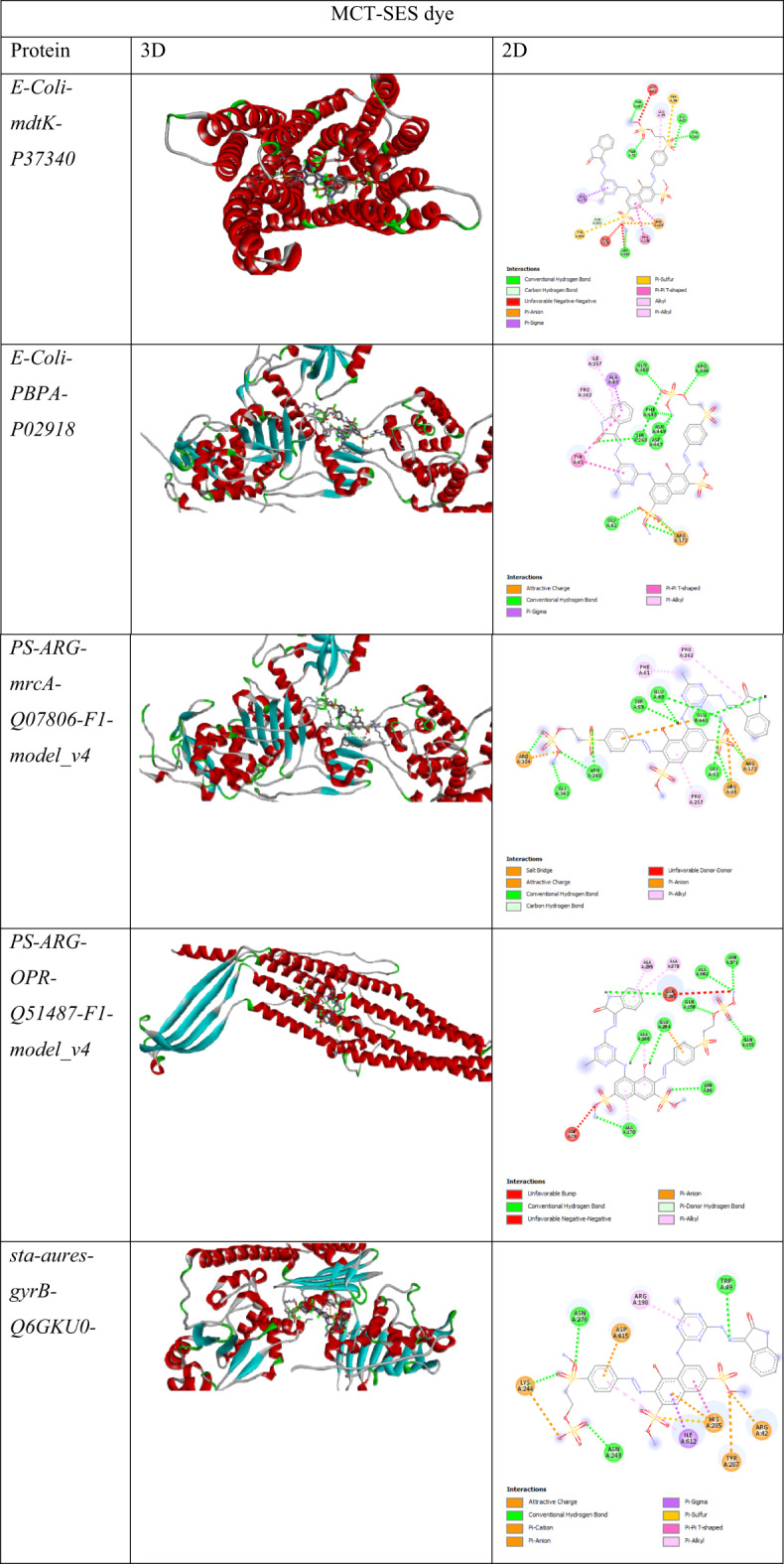

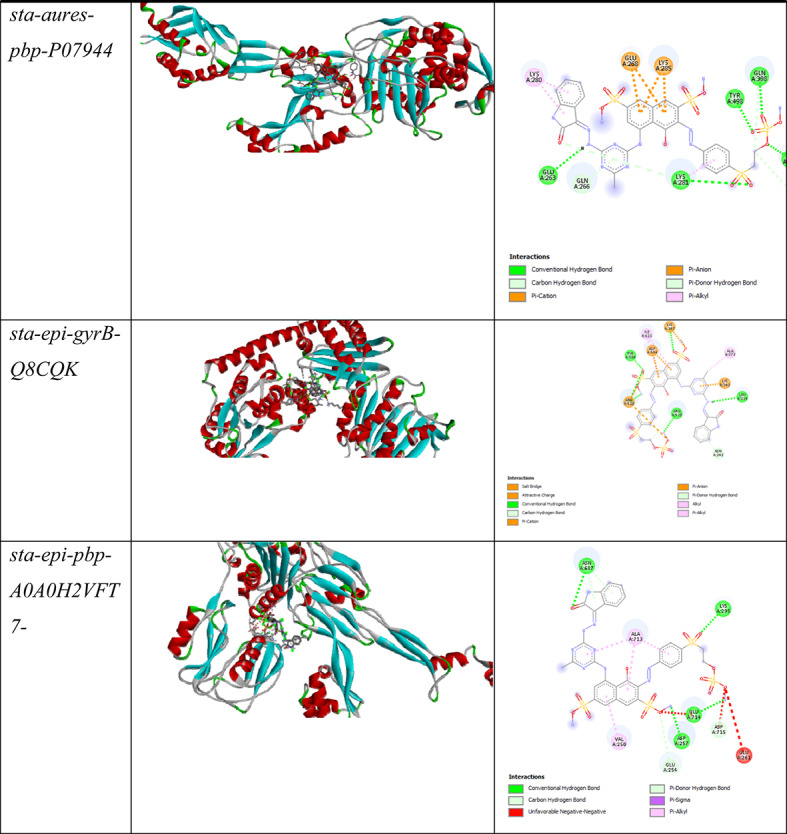
Comparative 3D and 2D structural visualizations of eight bacterial proteins.

**Table 7:** Summarizes of Docking scores and key interactions between MCT-SES dye and selected bacterial target proteins.

Receptor	Ligand	Docking Score
*E-Coli*-mdtK-P37340-F1-model_v4	MCT-SES dye	−10.2
*E-Coli*-PBPA-P02918-F1-model_v4	MCT-SES dye	−8.4
*PS-ARG*-mrcA-Q07806-F1-model_v4	MCT-SES dye	−8.2
*PS-ARG*-OPR-Q51487-F1-model_v4	MCT-SES dye	−9.7
*sta-aures*-gyrB-Q6GKU0-F1-model_v4	MCT-SES dye	−10.3
*sta-aures*-pbp-P07944-F1-model_v4	MCT-SES dye	−8.1
*sta-epi*-gyrB-Q8CQK4-F1-model_v4	MCT-SES dye	−10.2
*sta-epi*-pbp-A0A0H2VFT7-F1-model_v4	MCT-SES dye	−10.4

Table 7 provide valuable insights into the interaction patterns between MCT-SES dye and various bacterial proteins from *E. coli*, Pseudomonas aeruginosa, Staphylococcus aureus, and Staphylococcus epidermidis. These interactions reveal potential mechanisms of action and help elucidate structure-activity relationships that could guide future antimicrobial development efforts.

#### Binding affinity patterns

The docking scores obtained across all protein-ligand combinations demonstrated a consistent pattern where the MCT-SES dye compound exhibited strong binding affinity (−8.1 to −10.4 kcal/mol). This hierarchy of binding affinities suggests that structural features unique to the dye compound may be responsible for its enhanced interaction with bacterial targets. The consistently high binding scores across multiple bacterial species indicate that the MCT-SES dye compound may possess broad-spectrum antimicrobial potential, particularly against both Gram-positive (*S. aureus and S. epidermidis*) and Gram-negative (*E. coli and P. aeruginosa*) bacteria. Key Interactions with E. coli Proteins. The *E. coli* MdtK protein, which functions as a multidrug efflux pump, showed particularly strong interactions with MCT-SES dye dye ligand (−10.2 kcal/mol). Detailed analysis of these interactions revealed multiple conventional hydrogen bonds between the MCT-SES dye and key residues (GLY29, THR51, THR197, TYR201, and ASP368), suggesting a stable binding mode. The presence of π-π T-shaped interactions with PHE256 and PHE285 further stabilizes this complex. These interactions may effectively inhibit the efflux pump function, potentially allowing other antibiotics to accumulate within bacterial cells and exert their antimicrobial effects more efficiently. The *E. coli* PBPA, involved in peptidoglycan synthesis, similarly demonstrated strong interactions with the MCT-SES dye compound (−8.4 kcal/mol). The extensive network of hydrogen bonds with ARG172, SER260, GLN300, ARG304, PHE448, and ASN449 suggests that MCT-SES dye could interfere with the transpeptidase activity of PBPA, thereby disrupting cell wall synthesis. This mechanism is particularly important as it targets a bacterial-specific process not present in mammalian cells, potentially offering selectivity and reduced toxicity.

#### Pseudomonas aeruginosa target interactions

For *P. aeruginosa*, both the mrcA (encoding penicillin-binding protein 1) and OPR proteins displayed significant interactions with our compounds. The MCT-SES dye ligand showed strong binding to *P. aeruginosa* OPR (−9.7 kcal/mol), forming multiple hydrogen bonds with ASN86, GLN155, GLN159, and ALA302. The combination of these hydrogen bonds with pi-alkyl interactions with several alanine residues (ALA170, ALA166, ALA295, ALA378) creates a multifaceted binding mode that could effectively inhibit protein function. The interaction pattern with *P. aeruginosa* mrcA is particularly noteworthy, featuring complex salt bridge and attractive charge interactions involving ARG172, ARG304, and ARG65. These electrostatic interactions, complemented by conventional hydrogen bonds, create a binding environment that could significantly impair the enzymatic activity of this crucial cell wall synthesis protein. The presence of both electrostatic and hydrophobic interactions suggests an induced-fit mechanism of binding that could be challenging for the bacteria to overcome through simple mutations.

#### Staphylococcus species target interactions

The *S. aureus* and *S. epidermidis* proteins, particularly gyrB (DNA gyrase B subunit) and penicillin-binding proteins, demonstrated some of the strongest binding affinities with our compounds. The MCT-SES dye compound achieved binding scores of −10.3 kcal/mol with *S. aureus* gyrB and − 10.4 kcal/mol with S. epidermidis pbp. These exceptionally strong interactions suggest potential efficacy against these clinically important pathogens.

For *S. aureus* gyrB, the dye compound forms a complex network of interactions, including attractive charge interactions with ARG42 and LYS244, conventional hydrogen bonds with TRP49, ASN243, LYS244, and ASN276, and pi-based interactions involving HIS285. This diverse interaction profile targets the ATP-binding domain of DNA gyrase, potentially interfering with DNA replication and bacterial cell division. The involvement of both charged and aromatic residues in these interactions suggests that the dye compound’s mixed functional groups are well-suited for targeting this protein.

Similarly, the *S. epidermidis* proteins showed strong interactions with the dye compound, featuring numerous hydrogen bonds and hydrophobic interactions. The interaction with S. epidermidis pbp (−10.4 kcal/mol) exhibited hydrogen bonds with LYS295, ASN687, and electrostatic interactions with GLU714 and ASP257. These interactions target the catalytic domain of penicillin-binding proteins, potentially disrupting the Trans peptidase activity necessary for peptidoglycan cross-linking during cell wall synthesis.

#### Structure-activity relationship insights

The MCT-SES dye compound’s superior binding affinity can be attributed to its ability to form more extensive hydrogen bonding networks and engage in both electrostatic and hydrophobic interactions. The presence of aromatic rings in the MCT-SES dye structure facilitates pi-based interactions (π-π stacking, pi-alkyl, pi-anion) with aromatic and charged residues in the target proteins. The halogen atom (Cl) in the MCT-SES dye structure also participates in several interactions, contributing to the overall binding stability.

#### Biological activity implications

The consistent strong binding affinities observed with the dye compound across multiple bacterial species and protein targets suggest potential broad-spectrum antimicrobial activity. The targeting of multiple essential bacterial processes—efflux pumps (mdtK), cell wall synthesis (PBPA, mrcA, pbp), and DNA replication (gyrB)—indicates a possible multi-target mechanism of action that could reduce the likelihood of resistance development. The strong interactions with penicillin-binding proteins in both Gram-positive and Gram-negative bacteria suggest that the MCT-SES dye compound might function as a *β*-lactam mimetic, interfering with peptidoglycan synthesis in a manner similar to β-lactam antibiotics but potentially evading common resistance mechanisms such as *β*-lactamase production. Additionally, the ability to bind strongly to efflux pumps like mdtK may allow the dye to function as an efflux pump inhibitor, potentially restoring the efficacy of other antibiotics against resistant strains. The interaction with DNA gyrase (gyrB) in both *Staphylococcus* species points to another mechanism of action similar to fluoroquinolone antibiotics. However, the binding site and interaction pattern differ from traditional fluoroquinolones, suggesting potential activity against fluoroquinolone-resistant strains.

#### Integration with existing antimicrobial knowledge

These molecular docking results complement existing knowledge of antimicrobial targets and mechanisms. The targeting of penicillin-binding proteins aligns with the well-established efficacy of *β*-lactam antibiotics, while the interaction with DNA gyrase parallels the mechanism of fluoroquinolones. The potential inhibition of efflux pumps adds another dimension to the compounds’ activity profile, addressing one of the major mechanisms of antibiotic resistance. The multi-target approach evidenced by our docking studies aligns with emerging strategies in antimicrobial drug development, where targeting multiple essential bacterial processes simultaneously is seen as a promising approach to combat resistance. The structural insights gained from these docking studies provide a rational basis for further optimization of these compounds, potentially guiding medicinal chemistry efforts to enhance binding affinity, selectivity, and pharmacokinetic properties.

## Conclusion

According to our obtained results, it can be conclude that the prepared isatin based MCT-SES as multi-functional reactive dye exhibits high affinity for dyeing of cotton and wool fabrics. The prepared MCT-SES based isatin dye also shows high affinity as corrosion inhibitor, achieving a maximum of 98.64% at a concentration of 500 ppm. Additionally, this dye shows high performance affinity for bacteria inhibition towards Gram- positive and gram -negative bacteria through the interaction of the dye structure with the cell membrane, which led to the stunts growth of bacteria even on the dyed fabrics. This dye behavior is attributed to the chemical structure combination between the isatin and SES and MCT terminal reactive groups connected with H acid chromophore. Thus, amid NH and hydrazide NH, as well as the SES groups, interact for corrosion inhibition and act as active sites for bacteria inhibition. Additionally, the reactive groups play a main role in the high fixation of the dye molecules into the fabrics. Thus, this dye can be considered as a multifunctional material with high efficiency as corrosion inhibitor and antimicrobial dye against bacteria for medical applications.

## Data Availability

The datasets used and/or analyzed during the current study are available from the corresponding author upon reasonable request.
